# Telomerase Knockout in Myeloid Cells Predisposes Mice to Foam Cell Formation, Dyslipidemia, Lung Fibrosis, and Cardiac Dysfunction

**DOI:** 10.1111/acel.70490

**Published:** 2026-04-16

**Authors:** Zhanguo Gao, Yongmei Yu, David Wiggins, Eva M. Sevick‐Muraca, Mikhail G. Kolonin

**Affiliations:** ^1^ The Brown Foundation Institute of Molecular Medicine University of Texas Health Science Center Houston Texas USA

**Keywords:** atherosclerosis, cardiac, dyslipidemia, fibrosis, heart, inflammation, myeloid, senescence, telomerase, TERT

## Abstract

Aging‐associated changes in myeloid cells are incompletely understood. One of the culprits of aging is the downregulation of the *Tert* gene coding for the catalytic subunit of telomerase. Studies of mouse models with *Tert* knockout (KO) in specific cells have revealed the importance of the telomere‐independent noncanonical function of TERT in supporting mitochondrial metabolism and protection from cell senescence. To investigate the role of TERT in myeloid cells (MCs), we analyzed mice with *Tert* KO in the LysM+ lineage, indelibly labeled with membrane green fluorescent protein (mG). Macrophage numbers and properties in various organs were compared. Changes in the vasculature, adipose tissue (AT) depots, lungs, heart, and other tissues were assessed. MC‐*Tert‐*KO mice displayed MC depletion in the bone marrow and abnormal frequencies in other organs. *Tert‐*KO MCs were found to express senescence markers despite having a normal telomere length. *Tert*‐KO macrophages were found to be polarized toward the pro‐inflammatory M1 phenotype, upregulate expression of genes promoting lipid uptake and retention, and be prone to conversion into foam cells. MC‐*Tert‐*KO mice fed a high‐calorie diet had increased adiposity and dysfunctional glucose metabolism. On an atherogenic diet, MC‐*Tert‐*KO mice displayed abnormal lipid metabolism and chronic fever. Aged MC‐*Tert‐*KO mice developed pulmonary fibrosis and an imbalance in right/left ventricle cardiac output. Our results show that the increased conversion of *Tert*‐KO MCs into foam cells leads to systemic organ dysfunction. We conclude that TERT plays an important noncanonical function in MCs that prevents the development of aging‐associated diseases.

## Graphical abstract

Myeloid‐specific *Tert* knockout induces telomere‐independent senescence, pro‐inflammatory polarization, and foam cell formation, driving metabolic dysfunction and multi‐organ pathology. TERT thus plays a critical noncanonical role in myeloid cells in preventing aging‐associated diseases.

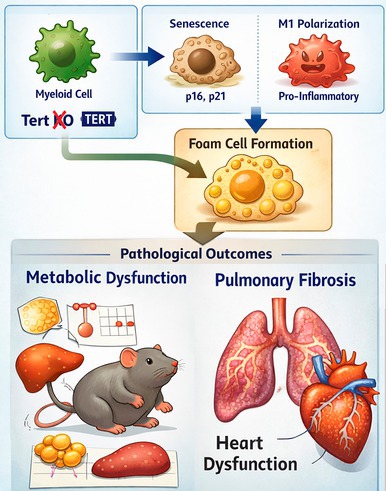

## 1 Introduction

Cell dysfunction, progressing during aging, is the foundation of metabolic and degenerative diseases. It is typically rooted in the accumulation of genotoxic and mitochondrial stress, often leading to cell senescence, the state of cell‐cycle arrest, and inflammatory cytokine secretion (Yousefzadeh et al. 2021). The repercussions of cell senescence in hematopoietic lineages are not completely understood. They are particularly important to consider for myeloid cells (MCs), the most abundant leukocytes that serve multipronged functions throughout the body. Macrophages and granulocytes, the primary monocyte types, play key roles in immunity as well as in aging‐linked disease development (Ng et al. 2023). Immuno‐senescence and specifically senescence of MCs has been linked with the syndrome of “homeostatic frailty”, a distinctive trait of the elderly, which predisposes to immune debilitation and chronic low‐grade inflammation, aka inflammaging (Bleve et al. 2023). Senescent MCs also promote extracellular matrix remodeling and fibrosis through cytokine secretion, impaired efferocytosis, and accumulation of iron reactive oxygen species (ROS) (Xiao et al. 2024). Aging and senescence of macrophages are linked with their polarization toward an M1‐like pro‐inflammatory phenotype (Liu et al. 2021) and excessive lipid accumulation, setting the stage for various organ pathologies (Wang et al. 2024). In metabolic disease, M1 macrophages have been shown to convert into foam cells, contributing to atherosclerotic plaques and aneurysm formation, the culprit of cardiovascular disease (Huang et al. 2015).

A molecular event predisposing to cell senescence is the inactivation of the *TERT* gene coding for the catalytic subunit of telomerase (Fossel et al. 2022). TERT is required for telomere maintenance, protection from oxidative stress, and mitochondrial function (Chakravarti et al. 2021). The dogma has been that TERT, active in stem cells, is turned off in somatic cells. However, it has become clear that TERT expression persists in many tissues both in mice and in humans. We have designed mouse models of tissue‐specific knockout (KO) of *Tert* in specific cell types (Rupert et al. 2025). We have reported that TERT KO in adipocyte progenitors accelerates telomere shortening, leads to cell senescence in adipose tissue (AT) of mice fed a high‐calorie diet, and accelerates type‐2 diabetes (T2D) development (Gao et al. 2020). We subsequently reported that *Tert* KO in endothelial and mesenchymal cells can cause cell dysfunction without telomere attrition (Gao et al. 2024). Our findings have demonstrated that the senescence phenotype due to TERT loss is linked with oxidative metabolism dysfunction and increased dependence on glycolysis. We have also demonstrated that transient ectopic expression of TERT and its noncanonical function in the mitochondria promotes oxidative cell metabolism (Gao et al. 2025). Hematopoietic cells are among the lineages normally retaining *TERT* expression through adulthood. This is evident from humans with *TERT* mutations being at increased risk for marrow failure (Yamaguchi et al. 2005). MCs have been specifically investigated and found to display a dynamic expression of *TERT* (Beyne‐Rauzy et al. 2005).

Here, we used the mouse model to test a hypothesis that TERT in MCs functions to suppress changes leading to age‐related pathologies. We show that *Tert* KO induces MC senescence and increases M1 macrophage polarization, promoting foam cell formation. We demonstrate that the abnormal MC function and organ distribution in MC‐Tert‐KO mice result in systemic metabolic abnormalities aggravated by high‐lipid diet feeding. Aging‐associated pulmonary fibrosis and cardiac ventricular output imbalance were revealed as the repercussions of MC dysfunction.

## 2 Materials and Methods

### 2.1 Animals and Ethical Approval

To generate mice with *Tert* KO specifically in myeloid cells, we crossed mice expressing Cre under the control of the LysM promoter (Clausen et al. 1999) with *TERT* ^fl/fl^ (Liu et al. 2015) and *mT/mG* (Muzumdar et al. 2007) mice. PCR‐based genotyping and analysis was performed as we previously described (Daquinag et al. 2015, 2011; Gao et al. 2020, 2018, 2022, 2024, 2023). *LysM‐cre*; *Tert* ^*fl/fl*^; *mTmG* (KO) and *LysM‐cre*; *Tert* ^*+/+*^; *mTmG* (WT) littermates were identified by PCR genotyping. All mice were housed in a 12‐h light/dark cycle in a temperature‐ and humidity‐controlled room, with ad libitum access to standard chow and water. Both the research team and the veterinary staff monitored animals daily. Animal health was monitored by weight (weekly), food and water intake, and general assessment of animal activity (daily). All animal studies were in compliance with the Guide and Use of Laboratory Animals and were approved by the UTHealth Animal Care and Use Committee.

### 2.2 Mouse Experiments

For obesity induction, mice were fed a high‐calorie (45 kcal% fat) diet (Research Diets D12451) from weaning until terminal tissue collection. For atherosclerosis induction, mice were fed a high‐cholesterol diet (Envigo TD.88137). Body composition was measured by EchoMRI‐100T (Echo Medical Systems). Glucose and insulin tolerance tests were performed as described in our previous studies (Gao et al. 2022, 2024). Indirect calorimetry data, food intake, and locomotor activity were quantified over a 3‐day time course in Phenomaster metabolic chambers (TSE Systems). Body temperature was measured in the rectum at 2.5 cm in depth using a MicroTherma 2K High Precision Type K Thermocouple Meter (ThermoWorks, THS‐221‐092) with RET‐3 probe (Braintree Scientific). Cold tolerance/adaptive thermogenesis was measured upon placing mice into the environmental chamber IS33SD (Powers Scientific) as described (Gao et al. 2020, 2018). Cardiac and respiratory gated CT scans were performed on mice following i.v. administration of 100–150 μL i.v. of nanoparticle contrast agent (Exitron Nano 12000, nanoPET Pharma) using an MILabs VECTor7CTUHR animal scanner (Houten, Netherlands), acquired with 0.5° step angle, 24 projections/step, and total scan time 18 min and 9 s. Images were processed using MILabs' reconstruction software at a 60 μm voxel size at the end exhale respiratory cycle and at 8 cardiac phases to find end systolic volume (ESV) and end diastolic volume (EDV) of both left and right ventricles. Analyses were performed blinded using Imalytics Preclinical 3.1 (Gremse‐IT). From ESV and EDV, ejection fraction % and cardiac output were then calculated using Stroke Volume (mm^3^) = EDV‐ESV, Ejection Fraction % = ((Stroke Volume)/EDV) ×100, Cardiac Output (mL/min) = (Stroke Volume × Heart Rate)/1000.

### 2.3 Cell Culture and Analysis

Bone marrow was collected from the femoral bones. Intraperitoneal macrophages were induced by ip injection of 2 mL of sterilized solution of 2% starch (Sigma, 85643) and harvested 3 days later with 10 mL of cold PBS in lavage from the i.p. cavity. AT cell suspensions were isolated as described (Gao et al. 2020, 2024). Minced tissue was digested in 0.5 mg/mL collagenase type I (Worthington Biochemical) and 2.5 mg/mL of dispase (Roche, 04942078001) solution under gentle agitation for 1 h at 37°C, filtered through 70 μm cell strainer (Fisherbrand, cat.# 22363548), and centrifuged at 400 g for 5 min to isolate the stromal/vascular pellet. Flow cytometry was performed as described (Gao et al. 2020) using Cytek Aurora and FlowJo with the following antibodies from Biolegend: Brilliant Violet 605 anti‐CD86 cat.# 105037, PE/FireTM700 anti‐CD206 cat.#141741, and APC‐anti‐F4/80 cat.# 123116. For cell culture, cells were plated in 8‐well chamber slides (Thermo Fisher, 154941) in DMEM/10% FBS. TelC‐Cy3 performed as described (Gao et al. 2024). After 4% paraformaldehyde fixation, 5 × 10^4^ cells/200 μL were probed with a telomere‐specific TelC‐Cy3 (PNA Bio, F1002) as described (Gao et al. 2020). For immunofluorescence (IF), the following antibodies were used: CD206 (R&D Systems, AF2534, 1:75); CD80 (Abclonal A23688, 1:50); CD68 (Invitrogen, MA5‐1324, 1:75); F4/80 (Abcam, ab16911, 1:50); TERT (Biossusa, bs‐1411R, 1:50). Where indicated, LPS (Sigma‐Aldrich, L2880) was added to the medium at 100 ng/mL for 240 min. Prior to Oil Red O staining (Sigma‐Aldrich, O0625) and BODIPY‐FL‐C_12_ (Invitrogen, D3822) uptake analysis, oxLDL (Athensresearch, 12‐16‐120412‐OX) was added to the medium at 25 μg/mL for 24 h.

### 2.4 Tissue Analysis

Plasma concentration of free fatty acids and HDL and LDL/VLDL cholesterol were measured with assay kits EnzyChrom EFA‐100 and AF E2HL‐100, respectively, from BioAssay System, as described by the manufacturer. SA‐βgal expression in tissue whole mounts was measured as described (Gao et al. 2020, 2018, 2024). AT formalin‐fixed paraffin‐embedded tissue sections (5 μm) were analyzed by IF upon antigen retrieval as described (Daquinag et al. 2015, 2011; Gao et al. 2018). Upon blocking, primary antibodies against F4/80 (abcam, ab16911, 1:75), CD206 (R&D, AF2534, 1:75), CD68 (Invitrogen, MA5‐1324, 1:75), Perilipin‐1 (Cell Signaling, 3470, 1:100), TNNT2 (Santa Cruz, sc‐20,025, 1:100), GFP (GeneTex, GTX22242, 1:200), RFP (Abcam, ab34771, 1:100) and CD31 (Santa‐Cruz, sc‐1506, 1:75) were applied (4°C, 12 h) followed by Donkey Alexa488‐conjugated (Invitrogen A11055, 1:200) or Cy3‐conjugated (Jackson ImmunoResearch, 711‐166‐152, 1:200) IgG. Nuclei were stained with Hoechst 33258 (Invitrogen, H3569). Fluorescence in situ hybridization (Telo‐FISH) was performed with TelC‐Cy3 as described (Gao et al. 2020, 2024). Briefly, slides treated with blocking reagent (Roche) were preheated for 5 min at 85°C followed by adding the TelC‐Cy3 probe (0.3 ng/mL) in hybridization buffer, incubation at 85°C for 10 min in a humidified chamber, and then hybridization at RT for 2 h. After 3 washes with PNA wash solution and PBS, samples counterstained with Hoechst were mounted in Fluoromount G. Images were acquired with a confocal Nikon AXR or Carl Zeiss upright Apotome Axio Imager Z1 microscope. Cell quantification was done using NIH ImageJ software by cell counts in 10 separate 10× fields. Analysis of aorta lesions was performed as described (Chen et al. 2022).

### 2.5 DNA and RNA Analyses

DNA was extracted as described (Gao et al. 2020, 2024) by the DNeasy Blood & Tissue Kit (QIAGEN, Cat. # 69504). A quantitative real‐time PCR (qRT‐PCR) method for absolute telomere length was used as described (Gao et al. 2020; O'Callaghan and Fenech 2011). RNA was extracted using the Trizol Reagent (Life Technologies, Cat. # 15596018). Complementary DNAs were generated using a High‐Capacity cDNA Reverse Transcription Kit (Applied Biosystems, Cat. # 4368814). PCR reactions were performed on CFX96 Real‐Time System C1000 Touch thermal cycler (Bio‐Rad) using Q‐PCR Master Mix (Gendepot, Cat. # Q5600‐005). qPCR reactions were set at 10 μL with 5 μL of the 2XMaster Mix, 0.8 μL primers, 3 μL of cDNA (15 ng), and 1.2 μL H_2_O. All qPCRs were run in duplicates on the same thermal cycles (95°C, 5 min, 40 cycles of 15 s at 95°C, 1 min at 60°C). Expression of mouse genes was normalized to *18S RNA*. Primer sequences ordered from Integrated DNA Technologies were as follows:

*Tert*: forward GGATTGCCACTGGCTCCG, reverse TGCCTGACCTCCTCTTGTGAC;

*p16/Ink*: forward TGCTCAACTACGGTGCAGATTC, reverse ATGTCTTGATGTCCCCGCTCT′;

*p21*: forward AACATCTCAGGGCCGAAA; reverse TGCGCTTGGAGTGATAGAAA;

*p53*: forward CGACCTATCCTTACCATCATCACA, reverse TTCTGTACGGCGGTCTCTCC;

*CoxIV*: forward CTGCCCGGAGTCTGGTAATG, reverse CAGTCAACGTAGGGGGTCATC;

*Tfam*: forward GGAATGTGGAGCGTGCTAAAA, reverse ACAAGACTGATAGACGAGGGG;

*Nrf1*: forward TATGGCGGAAGTAATGAAAGACG, reverse CAACGTAAGCTCTGCCTTGTT;

*Apoe*: forward CTCCCAAGTCACACAAGAACTG, reverse CCAGCTCCTTTTTGTAAGCCTTT;

*Ldlr*: forward GTGTGACCGTGAACATGACTGC, reverse CACTCCCCACTGTGACACTTGA;

*Abca1*: forward GGTTTGGAGATGGTTATACAATAGTTGT, reverse TTCCCGGAAACGCAA;

*Abcg1*: forward TTCATCGTCCTGGGCATCTT, reverse CGGATTTTGTATCTGAGGACGAA;

*Tnfa*: forward CTGTAGCCCACGTCGTAGC, reverse TTGAGATCCATGCCGTTG;

*Il1b*: forward TGTAATGAAAGACGGCACACC, reverse TCTTCTTTGGGTATTGCTTGG;

*Il6*: forward CAAAGCCAGAGTCCTTCAGA, reverse GATGGTCTTGGTCCTTAGCC;

*Mcp1*: forward ACTGAAGCCAGCTCTCTCTTCCTC, reverse TTCCTTCTTGGGGTCAGCACAGAC;

*Tgfb1*: forward CTCCACCTGCAAGACCAT, reverse CTTAGTTTGGACAGGATCTGG;

*Col1a1*: forward CCTCAGGGTATTGCTGGACAAC, reverse CAGAAGGACCTTGTTTGCCAGG;

*18s*: forward AAGTCCCTGCCCTTTGTACACA, reverse GATCCGAGGGCCTCACTAAAC.

### 2.6 Statistical Analysis

Power analysis and previous experience were used to guide the number of animals in each group to achieve statistical significance on the basis of the anticipated effects of the treatments and genetic manipulations. Data were analyzed using GraphPad Prism software. When normality assumptions were confirmed, groups were compared using homoscedastic Student *t*‐test for 2 groups or 1‐way or 2‐way ANOVA for ≥ 3 groups. *p* < 0.05 was considered significant. GraphPad Prism or Microsoft Excel was used to graph data as mean ± SEM. All experiments were repeated with similar results.

## 3 Results

### 3.1 *Tert* KO Indices Myeloid Cell Senescence Without Telomere Attrition

**FIGURE 1:**
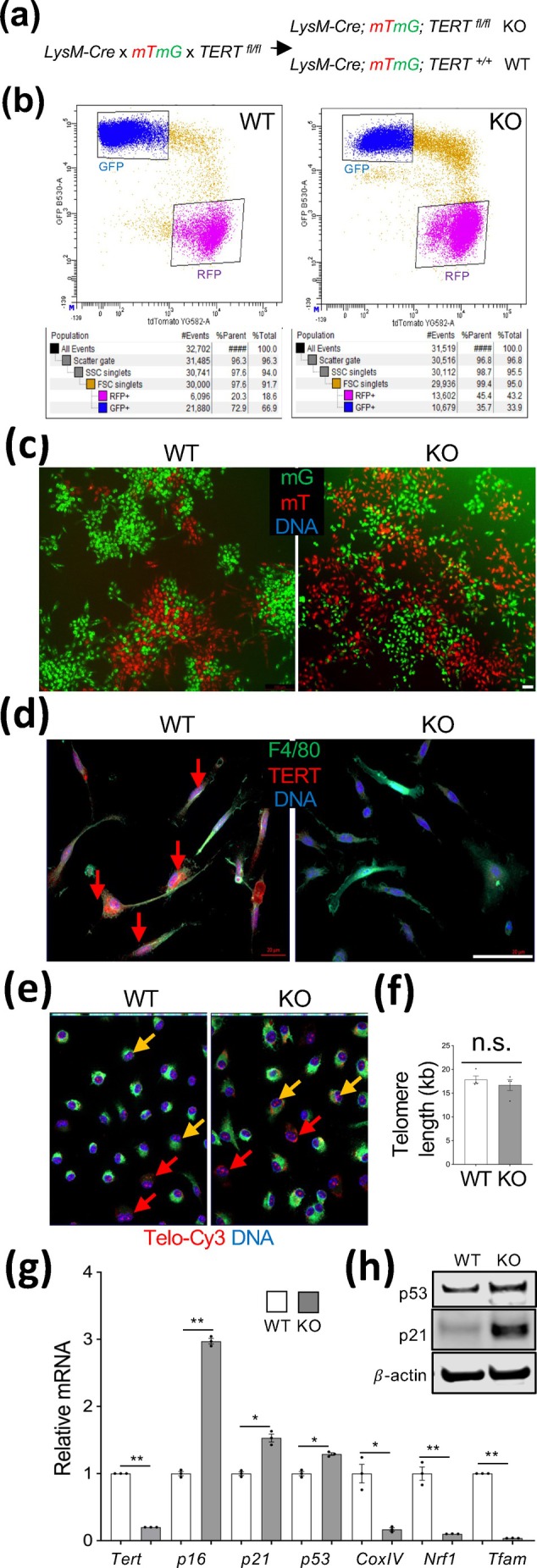
*Tert* KO indices myeloid cell senescence without telomere attrition. (a) Breeding scheme to generate mice with *Tert* KO in myeloid cells (MCs) of *LysM*+ lineage (KO) labeled with the mG reporter, among other cells expressing the mT reporter. (b) Flow cytometry on bone marrow cells from 21‐month‐old female mice, revealing a two‐fold lower mG+ cell frequency in KO. (c) Cells from (b) plated in primary culture demonstrating a lower mG+ cell frequency in KO. (d) mG+ cells from (b) in primary culture demonstrating a lack of TERT expression detectable by immunofluorescence in KO. (e) Telo‐FISH on adherent leukocytes reveals comparable telomere length (red TelC‐Cy3 signal) in mG+ cells (yellow arrows) and in mT+ cells (red arrows) from i.p. fluid of WT and KO mice. (f) A quantitative qPCR assay measuring the rate of telomeric DNA amplification relative to a single copy gene reveals a nonsignificant (n.s.) difference in telomere length in WT and KO intraperitoneal macrophages. (g) q‐RT‐PCR data (normalized to *18S* RNA) confirm *Tert* loss and reveal that senescence markers (*p16*, *p21*, and *p53*) are induced, whereas mitochondrial metabolism markers (*CoxIV*, *Nrf1*, and *Tfam*) are reduced in KO intraperitoneal macrophages. (h) Immunoblotting confirming the induction of p21 and p53 protein expression in KO intraperitoneal macrophages. For all data, mean+/− SEM (error bars). *p < 0.05, **p < 0.01 (two‐sided Student's *t*‐test). Scale bar: 50 μm.

To investigate the role of Tert in suppressing myeloid cell senescence, we used the *LysM* promoter specifically expressed in MC. We performed crosses to create *MC‐Tert*‐KO and control *LysM‐Cre* ^+^ mice also carrying the *mTmG* reporter (Figure 1a). This approach, used in our previous *Tert* KO studies (Gao et al. 2020, 2024, 2023, 2025), enables identification of MC as cells expressing membrane GFP (mG) among WT cells expressing membrane Tomato (mT). *LysM‐cre*; *Tert* ^*fl/fl*^; *mTmG* (KO) and *LysM‐cre*; *Tert* ^+^; *mTmG* (WT) littermates did not display apparent developmental differences. We analyzed littermates aged to 12 months, the age when mice with TERT KO in APC display markedly higher telomere attrition and metabolic dysfunction (Gao et al. 2020). Flow cytometric analysis of bone marrow revealed a reduction in the frequency of mG+ cells (MCs) in MC‐TERT‐KO mice (Figure 1b). While total immune (CD45+) and erythroid (TER119+) cell numbers were comparable, MC‐TERT‐KO mice had a lower frequency of lymphoid (CD3^+^) cells in the bone marrow (Figure S1a). The majority of bone marrow MCs are granulocytes, followed by monocytes/macrophages (Ng et al. 2023). Frequencies of total and of Ly6G+ granulocytes (neutrophils) were comparable in MC‐TERT‐KO and WT mice (Figure S1a). This indicated that the reduced mG+ frequency is largely due to monocyte depletion in the bone marrow.

In peripheral blood, total immune (CD45+) or erythroid (TER119+) cell numbers were also not affected by MC‐TERT‐KO. However, MC‐TERT‐KO mice had a much lower (5‐fold) frequency of total and of Ly6G+ granulocytes in peripheral blood (Figure S1b). Instead, MC‐TERT‐KO mice had a 7‐fold higher frequency of macrophages (F4/80+) in peripheral blood (Figure S1c). This provides further evidence for increased systemic recruitment of MCs for macrophage differentiation, which explains their depletion in the bone marrow.

Monocytes and macrophages adhere to plastic and can be cultured. Upon plating bone marrow cells, the reduced frequency of mG+ cells, relative to mT+ cells, was confirmed for MC‐TERT‐KO mice (Figure 1c). As expected, TERT‐KO mG+ cells adherent in culture lacked TERT expression (Figure 1d). For further analysis, we used intraperitoneal macrophages induced by starch injection. Telomere length was analyzed by fluorescent in situ hybridization (FISH) with a telomere probe. Telo‐FISH did not reveal a difference in telomere length in mG+ cells from KO and WT mice (Figure 1e). A quantitative PCR (qPCR) assay that measures the rate of telomeric DNA amplification relative to a single copy gene (O'Callaghan and Fenech 2011) confirmed that telomere length in WT and Tert‐KO MC was comparable (Figure 1f). Nevertheless, qRT‐PCR on mRNA from mG+ MC revealed that, along with the loss of *Tert*, a significant increase in senescence markers *p16*, *p21*, and *p53* was observed in KO MC (Figure 1g). Immunoblotting confirmed the induction of p21 (3‐fold) and p53 (1.7‐fold) expression at the protein level (Figure 1h). The larger size of mG+ KO cells observed in culture (Figure 1d) was also consistent with senescence induction. There was also a markedly decreased expression of a mitochondrial respiratory chain component, *CoxIV*, and of mitochondrial biogenesis markers, *Nrf1* and *Tfam* (Figure 1g). These results indicated that the mitochondrial function of TERT is jeopardized in Tert‐KO MC.

### 3.2 *Tert* KO Induces MCs to Convert Into Lipid‐Associated Macrophages and Foam Cells

**FIGURE 2:**
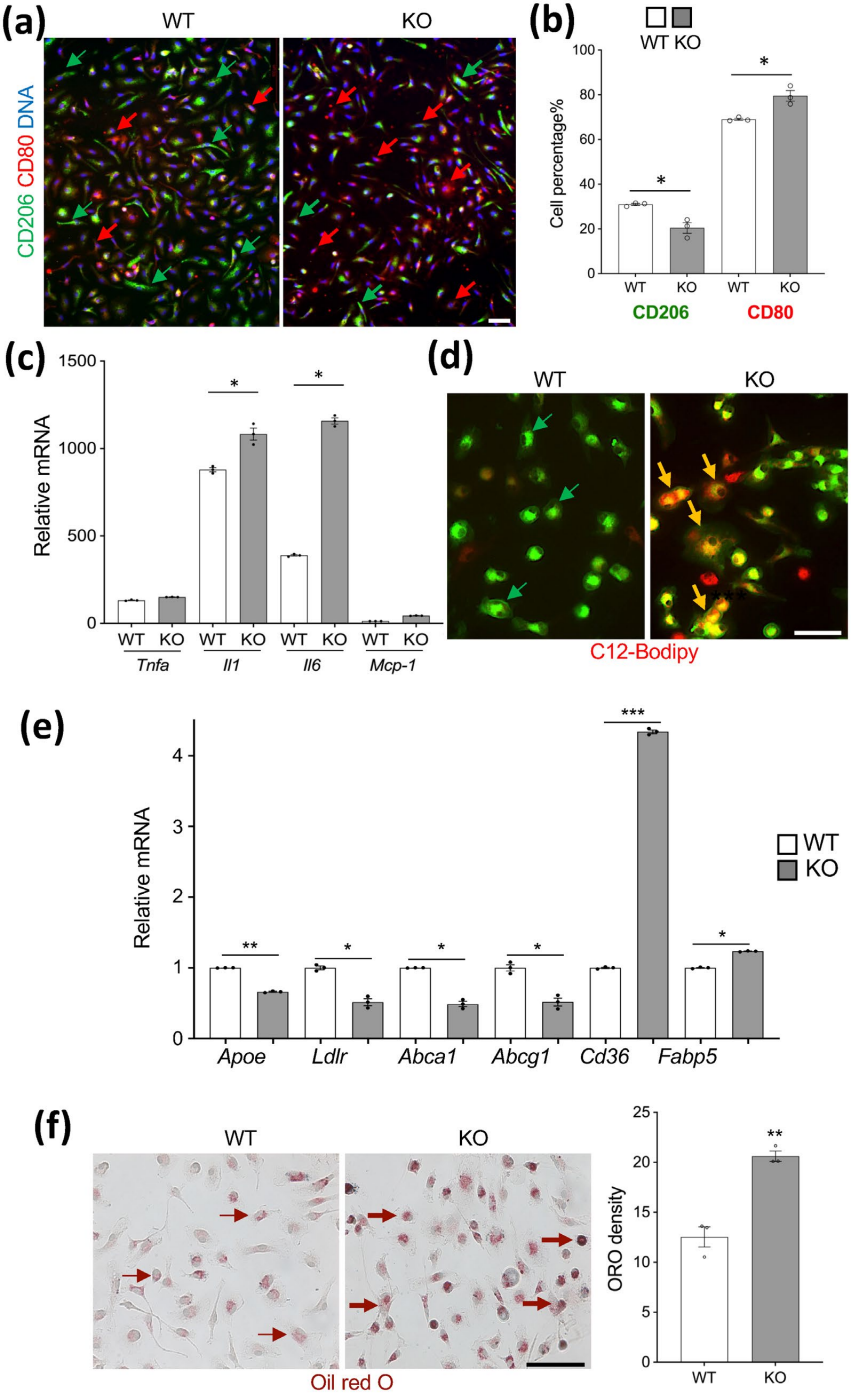
Tert KO induces lipid‐associated macrophages (LAMs). Starch‐induced i.p. macrophages from 2‐year‐old female mice were analyzed. (a) Upon adherence in primary culture, IF with antibodies against CD80 (M1‐macrophage) and CD206 (M2‐polarization) markers reveals a lower frequency of CD206^+^ macrophages in KO mice. (b) Data quantification from multiple fields of view in (a), indicating macrophage polarization shift toward the M1 phenotype. (c) Upon LPS (100 ng/mL, 4 h) treatment in primary culture, q‐RT‐PCR (normalized to *18S* RNA) demonstrates higher expression of genes coding for inflammation markers IL1 and IL6 in KO macrophages. (d) Macrophages were induced to convert into foam cells by oxLDL (0.025 mg/mL) treatment for 24 h. Note increased uptake of red‐fluorescent C_12_‐BODIPY (0.3 μM, 5 min) by mG+ KO cells (yellow arrows) compared to mG+ WT cells (green arrows) in primary culture. (e) q‐RT‐PCR (normalized to *18S* RNA) demonstrates lower expression of genes coding for lipid efflux effectors APOE, LDLR, ABCA1, ABCG1, and higher expression of genes coding for lipid transporters CD36 and FABP5 in KO oxLDL‐treated macrophages. (f) OxLDL‐treated macrophages stained with Oil Red O: Note larger lipid droplets (arrows) in KO cells. For all data, mean+/− SEM (error bars). *p < 0.05, **p < 0.01, ***p < 0.001 (two‐sided Student's *t*‐test). Scale bar: 50 μm.

Analysis of intraperitoneal macrophages in primary cell culture by immunofluorescence (IF) revealed that *Tert* KO resulted in a significant reduction in the frequency of cells expressing the M2 polarization marker CD206 (Figure 2a,b). The reciprocal increase in the frequency of cells positive for CD80 (Figure 2a,b) indicated a macrophage polarization shift toward the M1 phenotype. To confirm this, we analyzed the expression of inflammatory cytokines upon bacterial lipopolysaccharide (LPS) treatment of cultured cells. The expression of genes coding for IL1 and IL6 was induced to a significantly higher level in KO macrophages, compared to WT macrophages (Figure 2c). Because M1 polarization has been linked with increased lipid accumulation and conversion into foam cells, we tested the latter by treating cells with oxidized low‐density lipoprotein (oxLDL). The increased lipid uptake by KO macrophages was revealed upon incubation with a BODIPY‐labeled fatty acid (Figure 2d). We also analyzed the expression of genes regulating lipid transport by q‐RT‐PCR. This revealed increased expression of genes coding for proteins promoting cholesterol and fatty acid uptake (FABP5 and CD36) in KO macrophages (Figure 2e). In contrast, the expression of genes coding for proteins regulating cholesterol efflux, whose LOF mutations predispose to atherosclerosis (APOE, LDLR, ABCA1, and ABCG1) was reduced (Figure 2e). Consistent with this observation, upon oxLDL treatment, KO macrophages had higher triglyceride (TAG) content, as revealed by Oil Red O staining of lipid droplets (Figure 2f). These data indicated that *Tert* KO promotes MC conversion into lipid‐associated macrophages (LAMs). The larger size of KO macrophages observed in culture (Figure 2a,d,f) was also consistent with senescence induction.

### 3.3 Metabolic Dysfunction in MC‐*Tert*‐KO Mice on High‐Calorie Diet

**FIGURE 3:**
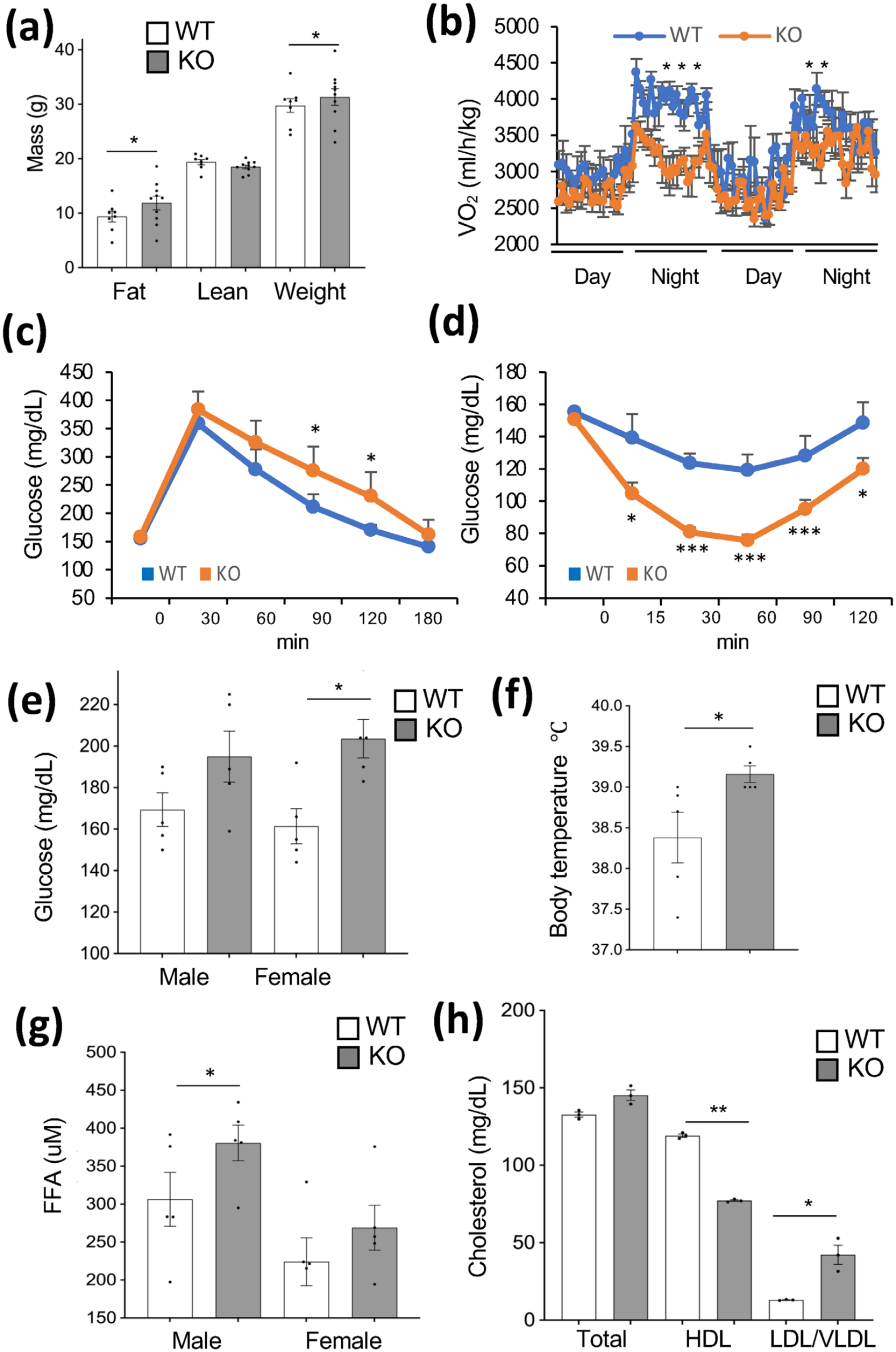
Metabolic dysfunction in LysM‐*Tert* KO mice. (a) Body composition of females fed a high‐calorie diet for 6 weeks. (b) Oxygen consumption in females after 6 weeks of high‐calorie diet feeding measured over 2 days/nights. (c) Glucose tolerance test after 6 months of high‐calorie diet feeding. (d) Insulin tolerance test after 6 months of high‐calorie diet feeding. (e) Non‐fasting glucose after 6 weeks of atherogenic diet feeding. (f) Body temperature after 12 weeks of atherogenic diet feeding. (g) Plasma free fatty acids after 12 weeks of atherogenic diet feeding. (h) Plasma LDL, HDL, and total cholesterol after 12 weeks of atherogenic diet feeding. High‐calorie diet: *N* = 5. Atherogenic diet: *N* = 5. For all data, mean +/− SEM (error bars). *p < 0.05, **p < 0.01, ***p < 0.001 (two‐sided Student's *t*‐test).

The observed macrophage phenotype is commonly linked with metabolic abnormalities. To assess that, we first analyzed mice raised on chow. There was no difference detected in body composition for KO versus WT mice (Figure S2a). To assess the effects of MC *Tert* KO in the state of positive energy balance, we compared metabolism in WT and KO mice fed a high‐calorie diet. While there was no difference in total body weight for KO versus WT mice, EchoMRI revealed that both KO females (Figure 3a) and males (Figure S2a) gained fat mass more rapidly than their WT littermates. That occurred despite food consumption being comparable for WT and KO mice (Figure S2b). Spontaneous locomotor activity was comparable in KO and WT mice (Figure S2c). Oxygen consumption was notably lower in female (Figure 3b) and male (Figure S2d) KO mice, compared to WT littermates, which accounts for increased fat mass accumulation in KO mice. KO mice fed a high‐calorie diet were found to be more glucose intolerant than WT littermates, which was the case for both females (Figure 3c) and males (Figure S2e). Interestingly, both female (Figure 3d) and male (Figure S2f) KO mice displayed higher insulin sensitivity, as well as lower HOMA‐IR (Figure S2g) than WT littermates. Glucose‐stimulated insulin secretion was lower in MC‐TERT‐KO mice, which provides an explanation for enhanced insulin sensitivity (Figure S2g). Both KO females and males had normal cold tolerance on a high‐calorie diet (Figure S2h).

### 3.4 Metabolic Dysfunction in MC‐*Tert*‐KO Mice on Atherogenic Diet

Pro‐inflammatory LAMs have been shown to convert to foam cells during atherogenesis (Moore et al. 2013). To determine the effect of *Tert* KO MCs on lipid metabolism in atherogenic conditions, we analyzed mice raised on a high‐cholesterol diet. Unlike for the high‐calorie diet, body composition was similar in WT and KO mice raised on an atherogenic diet (Figure S3a). Food consumption was comparable for WT and KO mice (Figure S3b). Nevertheless, higher glucose intolerance was still observed for both male and female KO mice fed an atherogenic diet (Figure S3c). Moreover, female KO mice had significantly higher non‐fasting plasma glucose than female WT mice (Figure 3e). No significant difference in the respiratory exchange ratio (RER) was observed (Figure S3d). Interestingly, unlike on the high‐calorie diet, KO mice were found to be in a state of low‐grade fever on the atherogenic diet (Figure 3f). Plasma analysis did not reveal a significant difference in circulating triglyceride levels between KO and WT littermates (Figure S3e). However, a trend for increased free fatty acid circulation was significant for male KO mice (Figure 3g). Also, significantly decreased levels of high‐density lipoprotein (HDL) and increased levels of very low‐density lipoprotein (VLDL) relative to LDL were observed in KO mice raised on an atherogenic diet (Figure 3h). Oil red O staining revealed areas of thickened aortic walls occasionally observed in KO mice (Figure S3f). However, atherosclerotic plaque build‐up has not been observed.

### 3.5 Adipose Tissue and Lung Fibrosis in MC‐*Tert*‐KO Mice

**FIGURE 4:**
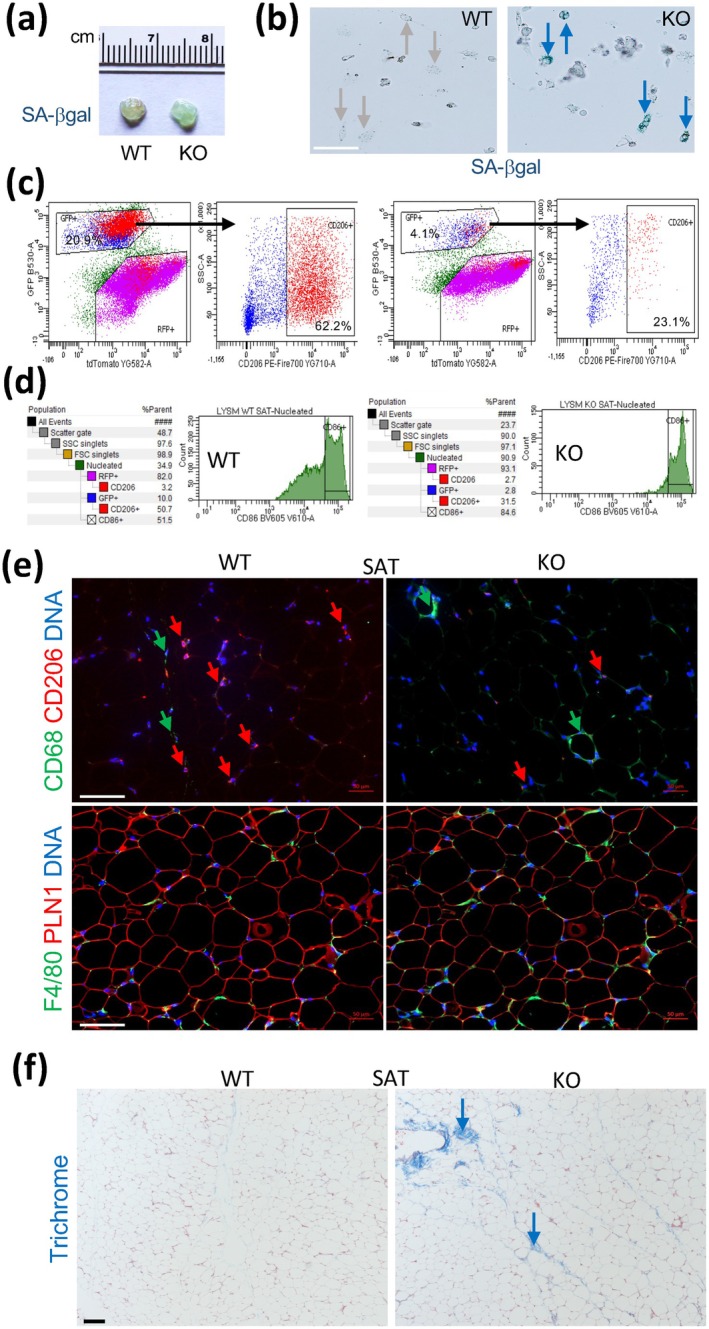
AT abnormalities in LysM‐*Tert* KO mice. (a) Senescence‐associated β‐galactosidase staining of VAT from 20‐month‐old female mice. (b) Senescence‐associated β‐galactosidase staining of adherent cells from VAT in (a). Arrows: Senescent cells. (c) Flow cytometry on VAT from A, revealing a lower frequency of mG+ macrophages expressing CD206 in KO mice. (d) Flow cytometry on SAT, revealing a higher frequency of mG+ macrophages expressing CD86 in KO mice. (e) IF with antibodies against CD68 and CD206 reveals a lower frequency of CD206+ macrophages (red arrows) in SAT of KO mice. IF with antibodies against perilipin‐1 and F4/80 reveals comparable adipocyte size in SAT of WT and KO mice. (f) Trichrome staining reveals fibrosis (arrows) in SAT of KO mice. In (d, e) 4‐month‐old male mice fed an atherogenic diet were used. Scale bar: 50 μm.

To look into the origin of the metabolic dysfunction, we compared AT depots in WT and KO mice. Consistent with the induction of *p16*, *p21*, and *p53* in KO MCs, analysis of AT revealed an increase in senescence‐associated beta‐galactosidase (SA‐βgal) activity in KO mice (Figure 4a). This was confirmed by SA‐βgal staining of primary cells derived from visceral adipose tissue (VAT) in cell culture (Figure 4b). To quantify, we analyzed AT by flow cytometry, which demonstrated a lower MC (mG+) frequency in both VAT (Figure 4c) and SAT (Figure 4d) of KO mice. Flow cytometric analysis for F4/80+ cells also indicated a reduction of macrophage frequency in VAT of MC‐Tert‐KO mice (Figure S1c). This was confirmed by IF on VAT sections (Figure S4a). In SAT, the overall numbers of F4/80+ macrophages revealed by flow cytometry (Figure S1c) and IF (Figure 4e) were found to be higher for MC‐Tert‐KO mice. This is likely to be due to relatively higher vascularization of SAT, compared to VAT, and circulating macrophages, increased by Tert‐KO, contributing to the readout.

The M2/M1 polarization of macrophages in AT was analyzed by flow cytometry. There was a three‐fold lower frequency of mG+ MCs expressing the M2 marker CD206 in VAT of MC‐Tert‐KO mice (Figure 4c). In SAT, the frequency of mG+CD206+ MCs was also lower (Figure 4d). Reciprocally, the frequency of mG+ macrophages expressing the M1 marker CD86 was higher in SAT of KO mice (Figure 4d). IF analysis of tissues from mice raised on HFD confirmed the reduced presence of M2‐polarized macrophages in both SAT (Figure 4e) and VAT (Figure S4b). In AT of KO mice, CD206‐negative macrophages tended to form more crown‐like structures, a hallmark of inflammation (Figure 4e; Figure S4b). Perilipin‐1 staining did not reveal an apparent effect of MC *Tert* KO on adipocyte size (Figure 4e, Figure S4b). Because M1 polarization and inflammatory cytokine secretion in AT have been linked with fibrosis (Misharin et al. 2017), we also stained AT with trichrome. This revealed increased collagen deposition in SAT of MC‐*Tert*‐KO mice (Figure 4f), indicating fibrosis development.

**FIGURE 5:**
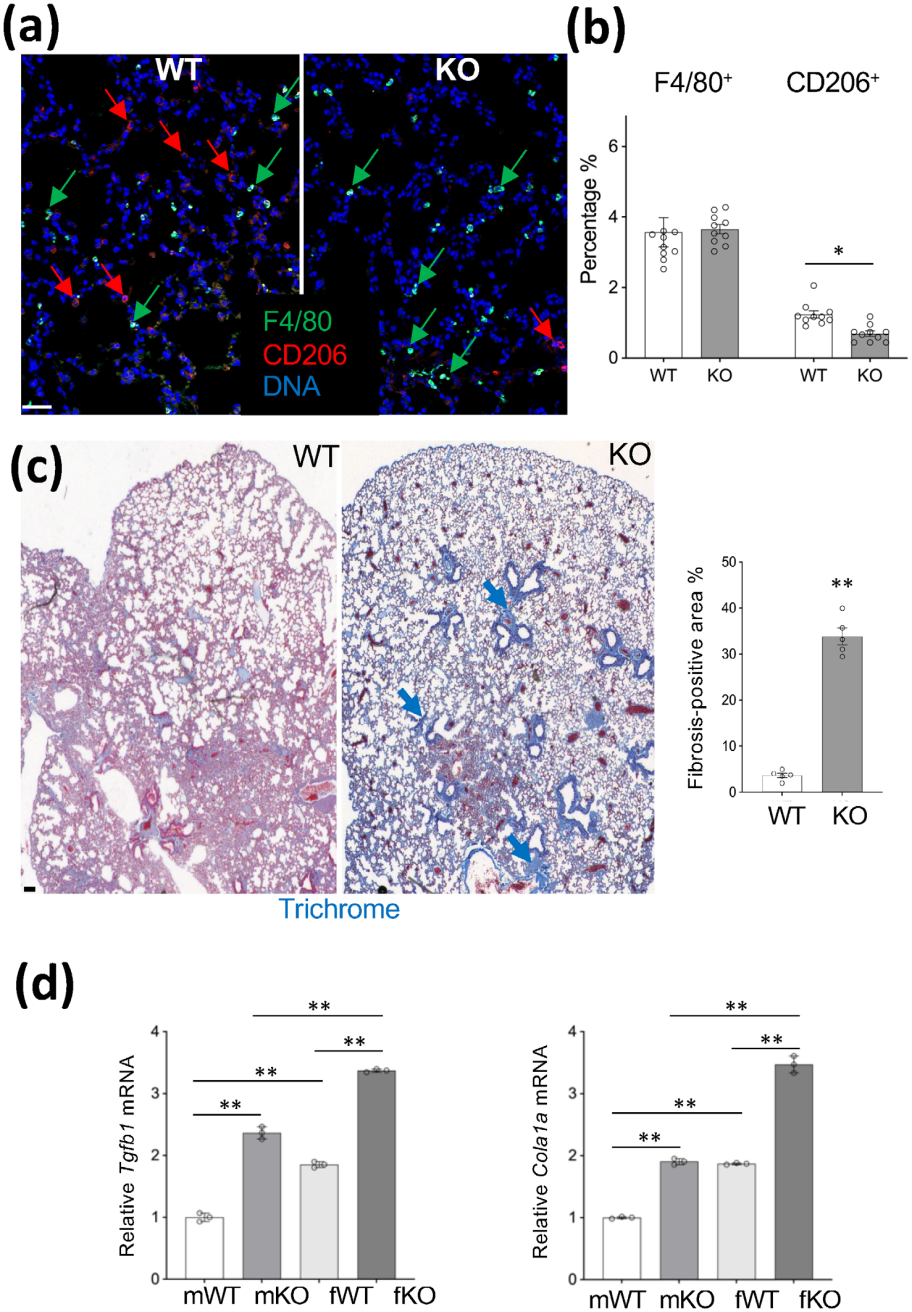
Lung abnormalities in LysM‐*Tert* KO mice. (a) IF with antibodies against F4/80 and CD206 reveals a lower frequency of CD206+ macrophages (red arrows) in lungs of 6 month‐old chow‐fed KO mice, compared to WT mice. (b) Data quantification for 10 view fields from A. *p < 0.05 (two‐sided Student's *t*‐test). (c) Trichrome staining reveals fibrosis (arrows) in 20‐month‐old lungs of KO mice. (d) q‐RT‐PCR (normalized to *18S* RNA) demonstrates higher expression of *Tgfb1* and *Cola1a* in lungs of KO male and female mice. Shown are mean+/− SEM (error bars). *p < 0.0001 (two‐sided Student's *t*‐test). ** *p* < 0.01. In (a), (b), and (d), 6‐month‐old male mice fed a chow diet were used. Scale bar: 50 μm.

Previous studies have shown that *Tert* KO fibroblasts convert to myofibroblasts, but their reduced proliferation and survival result in reduced pulmonary fibrosis (Liu et al. 2015). IF on lung tissue sections revealed that the overall numbers of macrophages (F4/80+) were not changed (Figure 5a,b). However, like in AT, the frequency of CD206+ macrophages was significantly lower in the lungs of KO mice compared to WT (Figure 5a,b). To determine the effect on the lungs, we stained sections with trichrome. This revealed an age‐dependent fibrosis induction in the lungs of KO mice, not observed at 6 months (Figure S5a) but profound in 20‐month‐old mice (Figure 5c). Consistent with this, the expression of *Tgfb*1, the master pro‐fibrotic factor, was significantly increased in the lungs of KO mice, alongside the induction of collagen *Col1a1*, a major extracellular matrix component (Figure 5d). Notably, the extent of fibrosis induction was more pronounced in female KO mice compared to males (Figure 5d). Analysis of liver sections revealed that, unlike in AT and the lung, MC (mG+ cell) infiltration was higher in KO mice than in WT mice (Figure S5b). However, there was no fibrosis induction observed in the liver (Figure S5c).

### 3.6 The Effect of MC‐*Tert* KO on Cardiac Function

**FIGURE 6:**
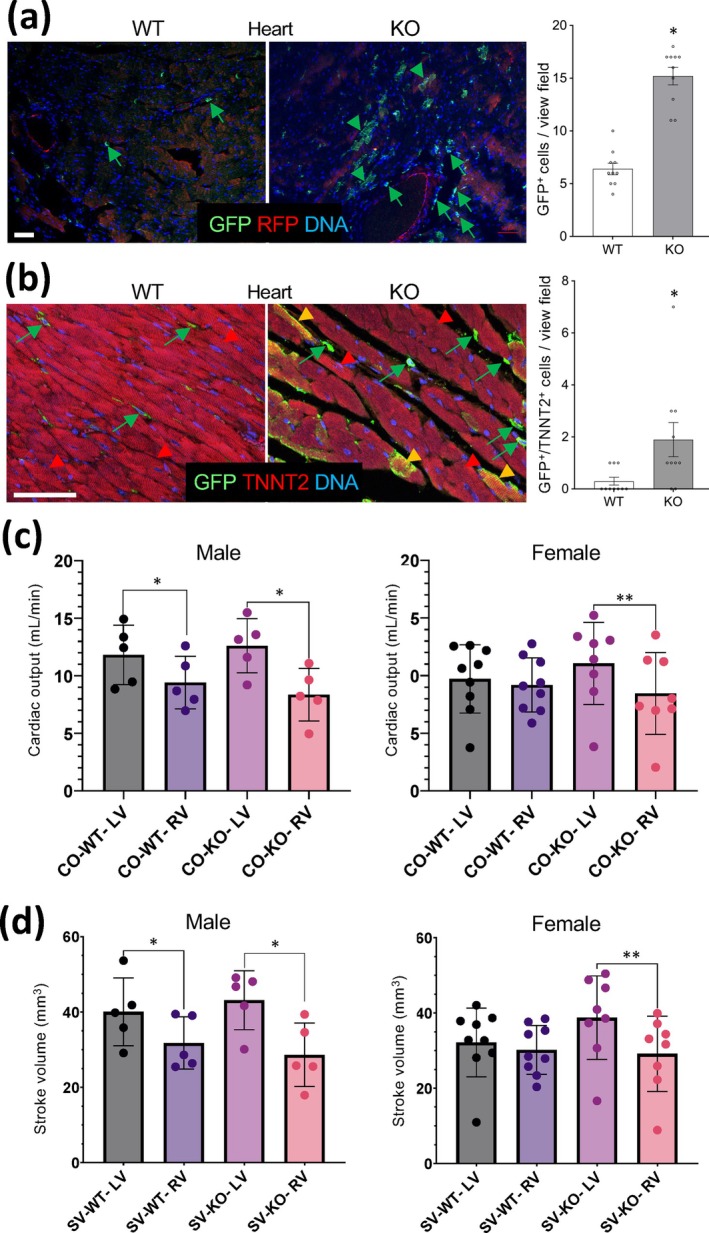
Heart abnormalities in LysM‐*Tert* KO mice. (a) Heart IF with antibodies against GFP and RFP reveals mG+ MCs (green arrows) in both WT and KO mice and striated mG+ cardiomyocytes (arrowheads) in KO mice. Graph: Mean +/− SEM from *N* = 10 view fields. (b) Heart IF with antibodies against TNNT2 and GFP shows cardiomyocytes (red arrowheads) and that some cardiomyocytes in KO mice express membrane GFP (yellow arrowheads). Green arrows: GFP+ MCs observed in both WT and KO mice. Graph: Mean +/‐SEM from *N* = 10 view fields. a, b: *p < 0.05 (homoscedastic Student's *t*‐test). Scale bar: 50 μm. c, d: Heart function analysis by CT (MILabs) plotted as mean +/− SD. (c) Cardiac output (CO) and (d) stroke volume (SV) quantifications for left ventricle (LV) and right ventricle (RV) indicate a disbalance in cardiac output. c, d: *p < 0.05; **p < 0.01 (one‐sided homoscedastic Student's *t*‐test).

There was no overt cardiac fibrosis observed in the heart of KO mice, according to trichrome staining (Figure S5d). Section analysis did not detect apparent differences in cardiac tissue histology between WT and KO mice (Figure S6a). However, higher numbers of mG+ cells were observed in the heart tissue of KO mice compared to WT mice (Figure 6a). In WT mice, MCs had intravascular localization, as expected (Figure 6a). Interestingly, in KO mice, in addition to luminal MCs, there were clusters of mG+ striated cells observed (Figure 6a). This was confirmed by co‐staining of cardiac tissues with antibodies against cardiac troponin T (TNNT2), a cardiomyocyte marker, high magnification of which reveals cardiomyocytes in KO mice expressing mG (Figure 6b). Expression of macrophage markers in these mG+/TNNT2+ cardiomyocytes was not observed (Figure S6b). In WT mice, only occasional mG+ cardiomyocytes were detected. This suggests that in the absence of *Tert*, MCs become prone to losing lineage specificity and mis‐differentiating into cardiomyocytes.

To determine if lungs and hearts abnormalities observed in KO mice have physiological implications, we compared statistically significant cohorts raised on HFD. Analysis of cardiac function was performed by gated cardiac CT imaging using gold nanoparticle contrast. There was no effect of MC‐Tert‐KO on heart rate (Figure S6c) or ejection fraction (Figure S6d) in either male or female mice. As expected, right and left ventricular stroke volumes and outputs were comparable in young (3‐month‐old) WT and KO mice (Figure S6e). In contrast, the ratio of right to left ventricle stroke volumes and outputs was decreased in 1‐year‐old KO mice fed HFD (Figure 6c,d). A relatively lower right ventricular cardiac output was also observed for 1‐year‐old WT males fed HFD (Figure 6c,d). Notably, this right/left disbalance was more pronounced in KO females than in KO males (Figure 6c,d). These data demonstrate that the organ response due to systemic *Tert*‐KO in MCs exacerbates abnormalities in cardiac function caused by high‐fat diet feeding.

## 4 Discussion

The onset of aging‐associated cell dysfunction is a driver of pathological tissue remodeling and disease. TERT has been shown to protect cells from senescence by enabling telomere maintenance, mitigating genotoxic stress, and supporting mitochondrial function (Fossel et al. 2022; Romaniuk et al. 2019; Sahin and Depinho 2010). Our recent report demonstrated an effect of *Tert* KO in MCs on cancer progression (Rupert et al. 2025). In the current study, we found that Tert KO in mouse MCs results in their senescence and dysfunction, characteristic of aging. MC‐Tert‐KO mice displayed MC depletion in the bone marrow and abnormal frequencies in extramedullary organs. We demonstrate that MC *Tert* knockout induces macrophage senescence and enhances M1 polarization, thereby promoting foam cell formation. Furthermore, we show that aberrant macrophage function and tissue distribution in *MC‐Tert*‐KO mice lead to systemic metabolic disturbances, which are exacerbated by high‐fat diet feeding. Aging‐associated pulmonary fibrosis and cardiac ventricle output imbalance were among the phenotypes we observed for *MC‐Tert*‐KO mice.

TERT has genome‐wide telomere‐independent effects on nuclear gene expression and cell metabolism (Mojiri et al. 2021). In addition, the extranuclear function of TERT in the mitochondria has been found as key to its geroprotective role (Ait‐Aissa et al. 2022; Ale‐Agha et al. 2021; Beyer and Norwood Toro 2018; Sahin et al. 2011). We have previously reported that the extranuclear function of TERT, supporting mitochondrial biogenesis and oxidative metabolism, was more important than its catalytic telomere extension activity in endothelial and mesenchymal cells (Gao et al. 2024, 2025). Our previous studies in mice with *Tert* KO in EC (Gao et al. 2024) and adipocytes (Gao et al. 2025) have demonstrated that metabolic abnormalities result from the lack of mitochondrial TERT function supporting respiration. Consistent with these reports, *Tert*‐KO MCs were found to have decreased expression of genes supporting oxidative metabolism. Importantly, this phenotype developed without increased telomere attrition. Our data suggest that MC senescence and metabolic abnormalities result from mitochondrial inadequacy. While there were sex‐specific differences observed in the *Tert* KO models reported previously (Gao et al. 2020, 2024), males and females had comparable phenotype expressivity in the current study. This suggests that the TERT‐supported function of MCs is less dependent on sex hormone regulation.

The *LysM* promoter is active in various MC populations, which needs to be taken into consideration in phenotype interpretation. Granulocytes, and particularly neutrophils, are abundant and known to underlie the pathogenesis of chronic pro‐inflammatory conditions. A mild decrease in granulocytes in peripheral blood (granulocytopenia) has been shown to protect against inflammatory remodeling and attenuate atherosclerosis progression and early‐stage lung fibrosis (Ding et al. 2021; Herro and Grimes 2024). Therefore, the observed reduction of granulocytes in circulation does not explain the functional changes observed in MC‐TERT‐KO mice.

Macrophages are known to exhibit dynamic functional shifts linked with metabolic rewiring. Increased aerobic glycolysis, altered fatty‐acid metabolism (oxidation and lipogenesis), mitochondrial dysfunction, disrupted amino‐acid and iron handling, as well as NAD^+^/redox imbalance program macrophages to adopt long‐lived proinflammatory/pro‐fibrotic phenotypes. These metabolically driven myeloid states (termed trained immunity) amplify lesion inflammation, promote fibroblast activation, and remodel cardiac/vascular ECM, producing progressive fibrosis and worse function (Riksen et al. 2023). They also impair resolution, the transition to an anti‐inflammatory, tissue‐repairing phenotype after the acute phase. Monocytes have been reported to directly transition to fibroblast‐like cells (fibrocytes), contributing to fibrogenesis (Pilling et al. 2009). However, proinflammatory MCs also act via cytokines and proteases to activate fibroblasts via paracrine signals (Misharin et al. 2017). We show that *Tert* KO macrophages produce more inflammatory cytokines upon LPS stimulation. The increased expression of *Tgfb1*, known to drive the deposition of extracellular matrix leading to tissue fibrosis, indicates the role of *Tert* KO MCs in the lung.

Lipid metabolism dysfunction is linked with the development of cardiovascular disease (Powell‐Wiley et al. 2021). We show that *Tert* KO MCs express high levels of FABP5 and CD36, the cholesterol/fatty acid transporter, and low expression of APOE, LDLR, ABCA1, and ABCG1, promoting cholesterol efflux. Expectedly, *Tert* KO MCs had features of LAMs and high efficiency of fatty acid uptake and conversion to foam cells when treated with cholesterol. LAMs, linked with metabolic disease, are known to drive the progression of atherosclerotic lesions (Tabas and Lichtman 2017). Foam‐cell formation due to increased cholesterol/oxLDL uptake reprograms macrophage lipid metabolism. In a vicious cycle, impaired cholesterol efflux and lysosomal dysfunction increase proinflammatory signaling. Fatty acid oxidation (FAO) and *de novo* lipogenesis can both be co‐opted: FAO supports anti‐inflammatory reparative functions in some contexts; however, dysfunctional FAO or lipid overload promotes ROS, inflammasome activation, and fibrotic cues in disease settings (Dai et al. 2024). M1 macrophages are predisposed to foam cell formation due to high lipid uptake and poor cholesterol efflux (Moore et al. 2013). Combined, our results indicate the increased propensity of *Tert*‐KO MC for conversion into foam cells, contributing to atherosclerosis. While some KO mice displayed aorta thickening, atherosclerotic plaque build‐up was not detected. Therefore, MC TERT‐KO is not sufficient to cause atherosclerosis. This is not surprising, as very few mutations cause plaque formation in mice without ApoE knockout.

Overall cardiac output in MC‐*Tert*‐KO and WT littermates was found to be comparable. However, MC‐*Tert*‐KO female mice fed HFD exhibited a significantly reduced right ventricular cardiac output relative to the left, despite a normal heart rate and ejection fraction. A growing 50% of heart failure in the U.S. is now recognized to result from heart failure with preserved ejection fraction (HFpEF), a condition where the right side of the heart fails despite a normal pumping of the left ventricle. This condition, for which there is little or no clinical management, often occurs because of pulmonary hypertension, which increases the workload on the right ventricle, leading to stiffness and dysfunction. Notably, HFpEF is particularly prevalent in aging women. Whether the cardiac phenotype selectively observed for MC‐*Tert*‐KO females is similar to the clinical condition of HFpEF remains to be further explored. Notably, the imbalance in cardiac output, developing with age in both WT and MC‐*Tert*‐KO males fed HFD, also became significant for aging MC‐*Tert*‐KO females fed HFD. This reiterates the notion that males are more prone to cardiovascular disease (Freedman et al. 2026) and indicates that *Tert*‐KO in MCs exacerbates diet‐induced abnormalities in cardiac function.

Resident cardiac MCs are the major immune cell type in the resting myocardium that act as key regulators of cardiac inflammation, tissue repair, and fibrosis. We observed increased numbers of MCs in the hearts of KO animals. Interestingly, cardiomyocytes derived from MCs were observed in KO but not WT littermates. This reduction in lineage commitment is akin to the conversion of Tert‐KO EC to adipocytes observed in our previous study (Gao et al. 2024). Whether this mis‐differentiation is observed clinically and if it contributes to cardiac dysfunction remains to be determined. Genetic mouse models of right heart disease are limited. In humans, mutations in the TGFβ pathway genes, leading to fibrosis and vascular remodeling, are linked with pulmonary arterial hypertension and right ventricular dysfunction. Our results are consistent with this and suggest that the MC‐*Tert*‐KO mice are a clinically relevant model of aging‐associated heart disease.

## 5 Conclusions

We conclude that TERT in MC enables normal mitochondrial metabolism, protection from cell senescence, as well as suppression of pro‐inflammatory macrophage polarization and foam cell formation. Metabolic abnormalities observed in MC‐*Tert*‐KO mice are an indirect consequence of the systemic changes in MC distribution and function. The important implication of our work is that pulmonary fibrosis and right heart failure can result merely from MC dysfunction upon TERT inactivation, which takes place during aging.

## Author Contributions

M.G.K. conceived and directed the project. Z.G., D.W., and Y.Y. performed the experiments and statistical analysis. Z.G., M.G.K., and E.M.S.‐M. analyzed and interpreted data. M.G.K. wrote the manuscript. Z.G. and E.M.S.‐M. reviewed and revised the manuscript. All authors discussed and approved the final manuscript.

## Funding

This work was supported by the NIH grant 1R01DK125922, the Levy‐Longenbaugh Fund, and the Bovay Foundation.

## Conflicts of Interest

The authors declare no conflicts of interest.

## Supporting information


**Figure S1:** Flow cytometry analysis of indicated organs from MC‐Tert‐KO versus WT chow‐fed females (12 months old). Antibodies used: APC rat anti‐mouse TER‐119 mAb (A27476), APC/Cyanine7 rabbit anti‐mouse CD45 mAb (A26831), PE rabbit anti‐mouse Ly‐6G mAb (A28037), and ABflo488 rat anti‐mouse CD3 mAb (A27161) from Abclonal. Cytek Aurora and FlowJo were used. In (c), the gates shown for peripheral blood were also used to quantify cells in VAT and SAT.

**Figure S2:** Metabolism of MC‐Tert‐KO versus WT mice fed chow for 4 months or a HFD for 6 weeks. (a) Body weight and body composition of males and females fed chow and of males fed HFD measured by EchoMRI. (b) HFD consumption by WT and KO mice. (c) Spontaneous locomotor activity of WT and KO mice post‐HFD. (d) Oxygen consumption by male WT and KO mice post‐HFD. (e) Glucose tolerance test in WT and KO males post HFD. (f) Insulin tolerance test in WT and KO males post‐HFD. (g) Males fed HFD were analyzed for glucose‐induced insulin secretion and HOMA‐IR (Fasting Insulin [μU/mL] × Fasting Glucose [mg/dL])/405. (h) Cold tolerance test in WT and KO mice post‐HFD. *N* = 5. For all data, mean+/− SEM (error bars). *p < 0.05, (two‐sided Student's *t*‐test).

**Figure S3:** Metabolic dysfunction in LysM‐Tert KO mice fed an atherogenic diet. Mice were analyzed after atherogenic diet feeding for 6 weeks. (a) Body composition of WT and KO male and female mice. (b) Atherogenic diet consumption by WT and KO mice. (c) Glucose tolerance test in WT and KO mice. (d) Respiratory exchange ratio (RER) in WT and KO mice. (e) Plasma triglycerides in WT and KO mice. For (a–e), *N* = 5. (f) Representative cross sections of aortic arch from WT and KO 1‐year‐old males, stained with hematoxylin/eosin. Scale bar: 100 μm. Arrow: representative wall thickening in KO mice. Measurement of wall thickness at *N* = 25 random cross‐section points for *N* = 5 mice is quantified by ImageJ, on the right. Plotted are mean+/− SEM (error bars). *p < 0.05, (two‐sided Student's *t*‐test).

**Figure S4:** AT abnormalities in LysM‐Tert KO mice. (a) IF with antibodies against GFP and RFP reveals a lower frequency of mG+ lineage cells (arrows) in VAT of KO male mice fed an atherogenic diet for 12 weeks. (b) IF with antibodies against CD68 and CD206 reveals a lower frequency of CD206+ macrophages (red arrows) in VAT of KO mice. IF with antibodies against perilipin‐1 and F4/80 reveals comparable adipocyte size in SAT of WT and KO mice. 4‐month‐old male mice fed an atherogenic diet were analyzed. Scale bar: 50 μm. (c) Flow cytometry analysis of VAT from MC‐Tert‐KO versus WT chow‐fed females (12 months old). Antibodies used: Cytek Aurora and FlowJo were used.

**Figure S5:** Organ fibrosis in LysM‐Tert KO mice. Fixed sections from mice of the indicated ages fed chow were analyzed. (a) Trichrome staining reveals a lack of excessive fibrosis in the lungs of 6‐month‐old KO mice. (b) IF analysis reveals a higher frequency of mG+ macrophages (green arrows) in the liver of 12‐month‐old KO mice. (c) Trichrome staining reveals a lack of excessive fibrosis in the livers of 12‐month‐old KO mice. (d) Trichrome staining reveals a lack of excessive fibrosis in the hearts of 12 month‐old KO mice. Scale bar: 50 μm. Graphs: data quantification (mean +/− SEM) for a–d. *N* = 5. *p < 0.05 (two‐sided Student's *t*‐test).

**Figure S6:** Heart analysis. (a, b) 1‐year‐old WT and KO male mice fed atherogenic diet. (a) Hematoxylin/eosin of heart sections. (b) Heart sections subjected to IF with F4/80 antibodies. Arrows: increased frequency of macrophages in KO mice. IB4: isolectin B4 marking the endothelium. Scale bar: 50 μm. Graphs: data quantification (mean+/− SEM) for a‐d. *N* = 5. *p < 0.05 (two‐sided Student's *t*‐test). (c, d) Mice fed HFD for 1 year (also analyzed in Figure 6c,d). (c) Heart rate. (d) Ejection fraction (EF). (e) Cardiac output (CO) and stroke volume (SV) in 3 month‐old male mice fed chow. LV, left ventricle; RV, right ventricle.

## Data Availability

The data that support the findings of this study are available on request from the corresponding author. The data are not publicly available due to privacy or ethical restrictions.
