# Correction to “Telomerase Knockout in Myeloid Cells Predisposes Mice to Foam Cell Formation, Dyslipidemia, Lung Fibrosis, and Cardiac Dysfunction”

**DOI:** 10.1111/acel.70619

**Published:** 2026-07-01

Gao, Z., Y. Yu, D. Wiggins, E. M. Sevick‐Muraca, and M. G. Kolonin. 2026. “Telomerase Knockout in Myeloid Cells Predisposes Mice to Foam Cell Formation, Dyslipidemia, Lung Fibrosis, and Cardiac Dysfunction.” *Aging Cell* 25: e70490. https://doi.org/10.1111/acel.70490.

In Figure 4e, the F4/80 PLN1 DNA image for WT is a duplicate of the F4/80 PLN1 DNA image for KO. The correct Figure 4 is shown below.

**Figure:**
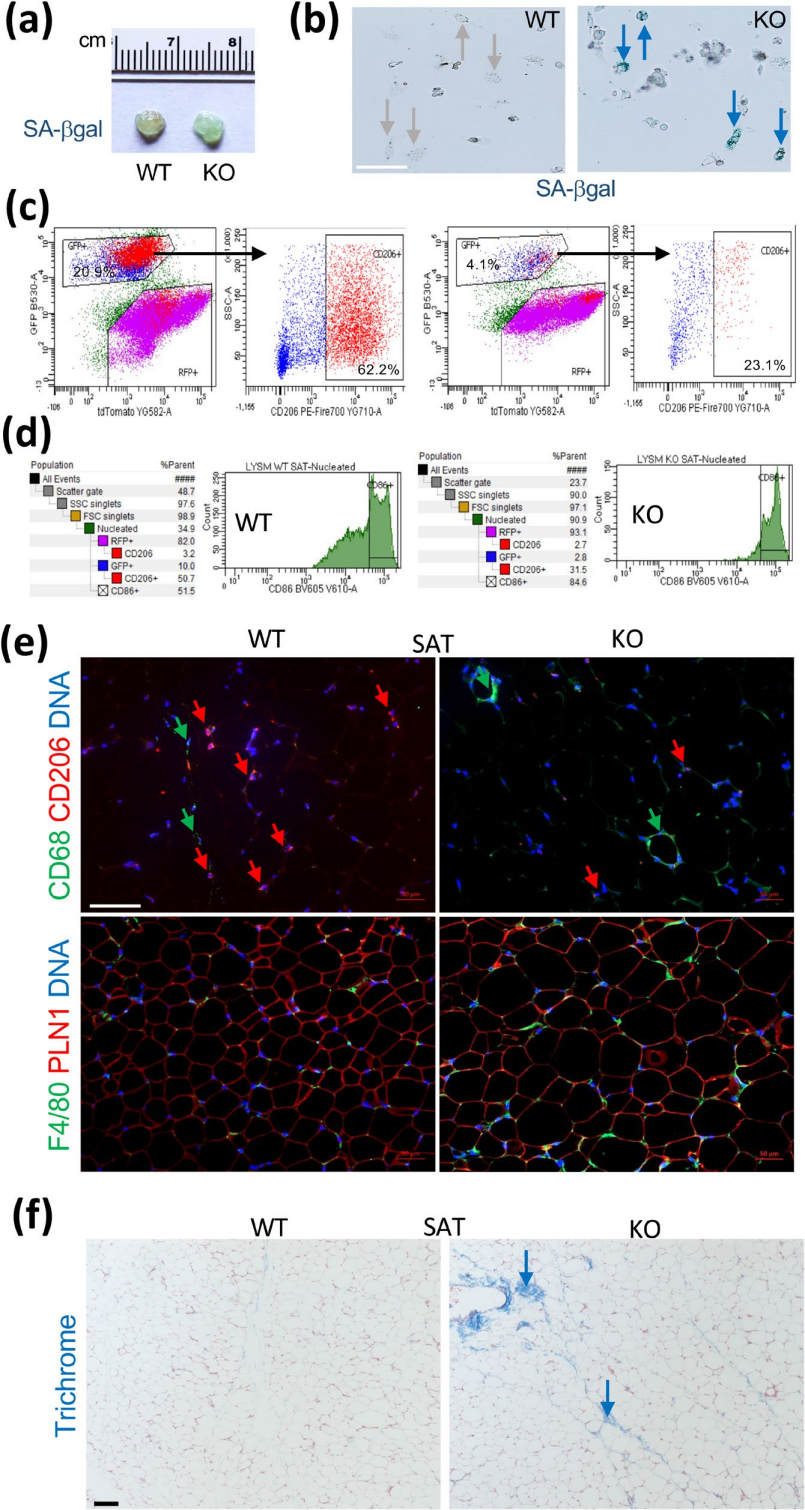
FIGURE 4. AT abnormalities in LysM‐*Tert* KO mice. (a) Senescence‐associated β‐galactosidase staining of VAT from 20‐month‐old female mice. (b) Senescence‐associated β‐galactosidase staining of adherent cells from VAT in (a). Arrows: Senescent cells. (c) Flow cytometry on VAT from A, revealing a lower frequency of mG+ macrophages expressing CD206 in KO mice. (d) Flow cytometry on SAT, revealing a higher frequency of mG+ macrophages expressing CD86 in KO mice. (e) IF with antibodies against CD68 and CD206 reveals a lower frequency of CD206+ macrophages (red arrows) in SAT of KO mice. IF with antibodies against perilipin‐1 and F4/80 reveals comparable adipocyte size in SAT of WT and KO mice. (f) Trichrome staining reveals fibrosis (arrows) in SAT of KO mice. In (d, e) 4‐month‐old male mice fed an atherogenic diet were used. Scale bar: 50 μm.

We apologize for this error.

