# Supplementary material for: Telomerase Knockout in Myeloid Cells Predisposes Mice to Foam Cell Formation, Dyslipidemia, Lung Fibrosis, and Cardiac Dysfunction
Source: Aging Cell. 2026 Apr 16;25(4):e70490. doi: 10.1111/acel.70490 (PMC13086613; doi:10.1111/acel.70490)
Supplement: Supplementary file 1 — Figure S1: Flow cytometry analysis of indicated organs from MC‐Tert‐KO versus WT chow‐fed females (12 months old). Antibodies used: APC rat anti‐mouse TER‐119 mAb (A27476), APC/Cyanine7 rabbit anti‐mouse CD45 mAb (A26831), PE rabbit anti‐mouse Ly‐6G mAb (A28037), and ABflo488 rat anti‐mouse CD3 mAb (A27161) from Abclonal. Cytek Aurora and FlowJo were used. In (c), the gates shown for peripheral blood were also used to quantify cells in VAT and SAT. [file ACEL-25-e70490-s006.pdf]

(a)

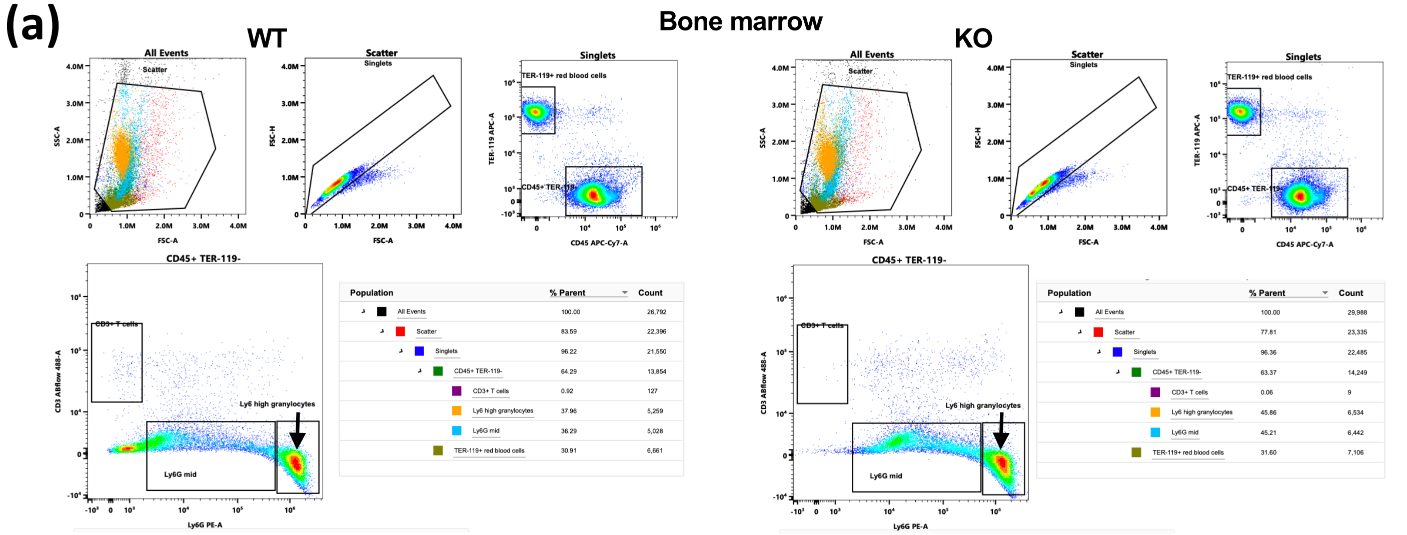

WT

Population	% Parent	Count
All Events	100.00	26,792
Scatter	83.59	22,396
Singlets	96.22	21,550
CD45+ TER-119-	64.29	13,854
CD3+ T cells	0.92	127
Ly6 high granylocytes	37.96	5,259
Ly6G mid	36.29	5,028
TER-119+ red blood cells	30.91	6,661

KO

Population	% Parent	Count
All Events	100.00	29,988
Scatter	77.81	23,335
Singlets	96.36	22,485
CD45+ TER-119-	63.37	14,249
CD3+ T cells	0.06	9
Ly6 high granylocytes	45.86	6,534
Ly6G mid	45.21	6,442
TER-119+ red blood cells	31.60	7,106

(b)

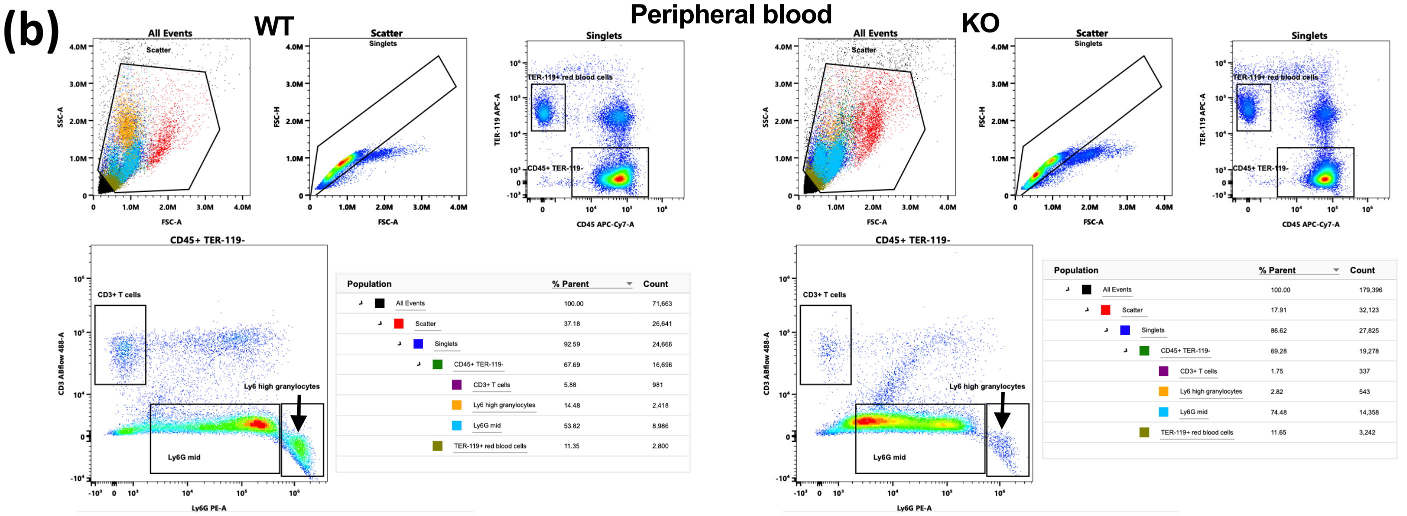

WT

Population	% Parent	Count
All Events	100.00	71,663
Scatter	37.18	26,641
Singlets	92.59	24,666
CD45+ TER-119-	67.69	16,696
CD3+ T cells	5.88	981
Ly6 high granylocytes	14.48	2,418
Ly6G mid	53.82	8,986
TER-119+ red blood cells	11.35	2,800

KO

Population	% Parent	Count
All Events	100.00	179,396
Scatter	17.91	32,123
Singlets	86.62	27,825
CD45+ TER-119-	69.28	19,278
CD3+ T cells	1.75	337
Ly6 high granylocytes	2.82	543
Ly6G mid	74.48	14,358
TER-119+ red blood cells	11.65	3,242

(c)

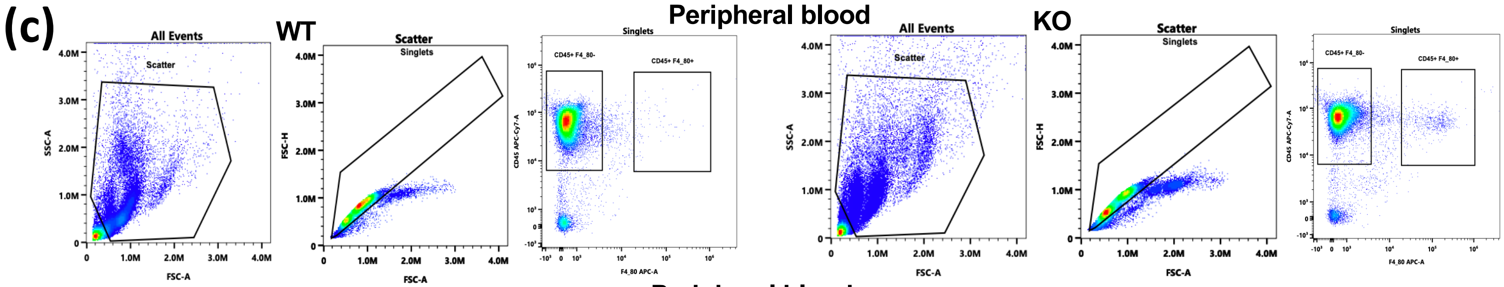

Peripheral blood

WT

Population	% Parent	Count
All Events	100.00	56,390
Scatter	41.81	23,579
Singlets	91.25	21,515
CD45+ F4_80-	85.99	18,501
CD45+ F4_80+	0.36	77

KO

Population	% Parent	Count
All Events	100.00	135,311
Scatter	19.52	26,414
Singlets	80.66	21,305
CD45+ F4_80-	85.48	18,211
CD45+ F4_80+	2.79	594

VAT

WT

Population	% Parent	Count
All Events	100.00	1,664
Scatter	38.70	644
Singlets	87.58	564
CD45+ F4_80-	5.14	29
CD45+ F4_80+	2.48	14

KO

Population	% Parent	Count
All Events	100.00	4,952
Scatter	47.90	2,372
Singlets	93.34	2,214
CD45+ F4_80-	3.93	87
CD45+ F4_80+	0.95	21

SAT

WT

Population	% Parent	Count
All Events	100.00	5,240
Scatter	46.15	2,418
Singlets	93.22	2,254
CD45+ F4_80-	25.60	577
CD45+ F4_80+	1.24	28

KO

Population	% Parent	Count
All Events	100.00	3,208
Scatter	38.84	1,246
Singlets	91.65	1,142
CD45+ F4_80-	14.89	170
CD45+ F4_80+	3.42	39

Figure S1 Flow cytometry analysis of indicated organs from MC-*Tert*-KO vs WT chow-fed females (12 months old). Antibodies used: APC rat anti-mouse TER-119 mAb (A27476), APC/Cyanine7 rabbit anti-mouse CD45 mAb (A26831), PE rabbit anti-mouse Ly-6G mAb (A28037), and ABflo488 rat anti-mouse CD3 mAb (A27161) from Abclonal. Cytek Aurora and FlowJo were used. In (c), the gates shown for peripheral blood were also used to quantify cells in VAT and SAT.
