# Supplementary material for: Telomerase Knockout in Myeloid Cells Predisposes Mice to Foam Cell Formation, Dyslipidemia, Lung Fibrosis, and Cardiac Dysfunction
Source: Aging Cell. 2026 Apr 16;25(4):e70490. doi: 10.1111/acel.70490 (PMC13086613; doi:10.1111/acel.70490)
Supplement: Supplementary file 2 — Figure S2: Metabolism of MC‐Tert‐KO versus WT mice fed chow for 4 months or a HFD for 6 weeks. (a) Body weight and body composition of males and females fed chow and of males fed HFD measured by EchoMRI. (b) HFD consumption by WT and KO mice. (c) Spontaneous locomotor activity of WT and KO mice post‐HFD. (d) Oxygen consumption by male WT and KO mice post‐HFD. (e) Glucose tolerance test in WT and KO males post HFD. (f) Insulin tolerance test in WT and KO males post‐HFD. (g) Males fed HFD were analyzed for glucose‐induced insulin secretion and HOMA‐IR (Fasting Insulin [μU/mL] × Fasting Glucose [mg/dL])/405. (h) Cold tolerance test in WT and KO mice post‐HFD. N = 5. For all data, mean+/− SEM (error bars). *p < 0.05, (two‐sided Student's t‐test). [file ACEL-25-e70490-s001.pdf]

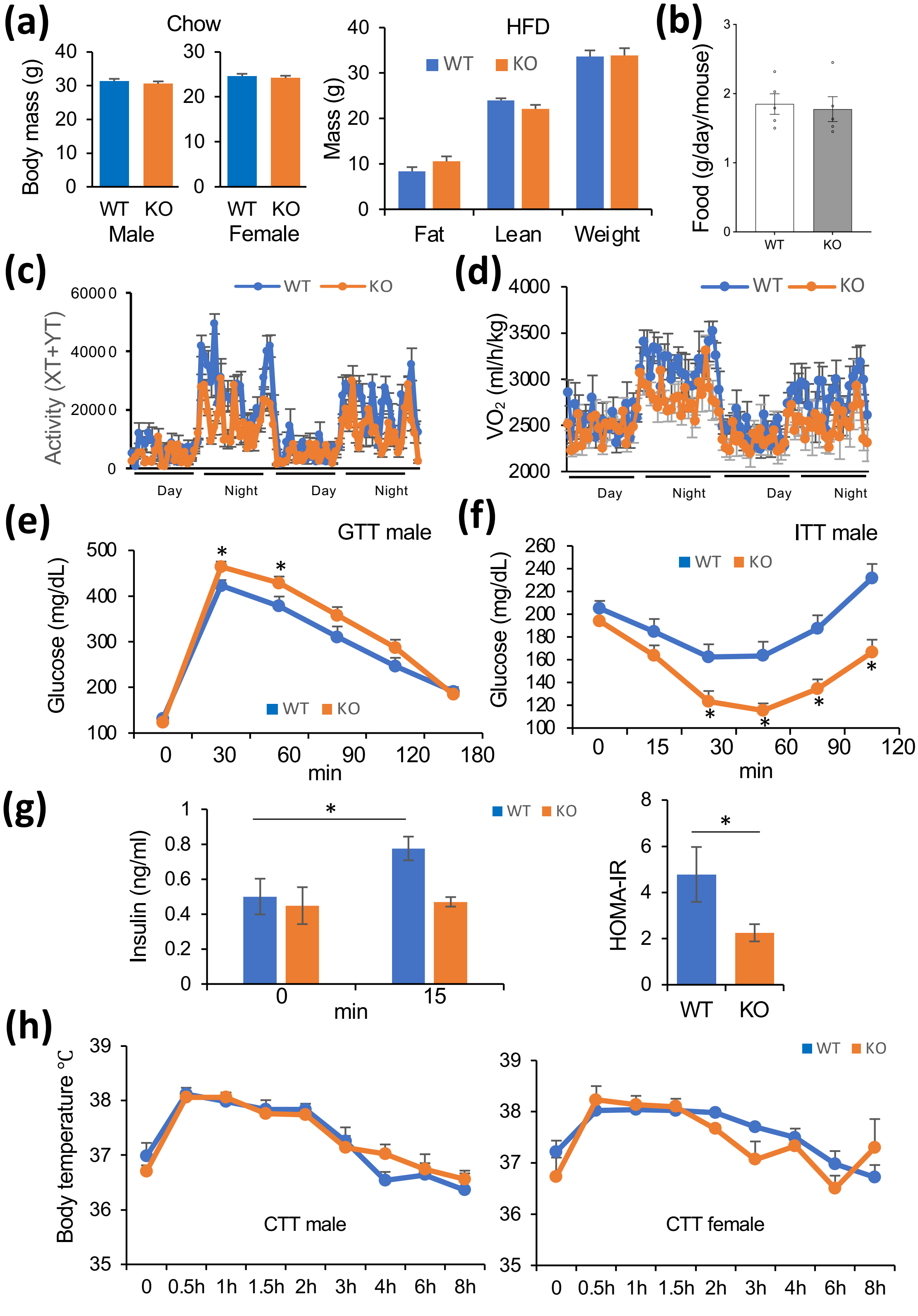

Figure S2 Metabolism of MC-*Tert*-KO vs WT mice fed chow for 4 months or a HFD for 6 weeks. (a) Body weight and body composition of males and females fed chow and of males fed HFD measured by EchoMRI. (b) HFD consumption by WT and KO mice. (c) Spontaneous locomotor activity of WT and KO mice post-HFD. (d) Oxygen consumption by male WT and KO mice post-HFD. (e) Glucose tolerance test in WT and KO males post-HFD. (f) Insulin tolerance test in WT and KO males post-HFD. (g) Males fed HFD were analyzed for glucose-induced insulin secretion and HOMA-IR (Fasting Insulin [μU/mL] × Fasting Glucose [mg/dL]) / 405. (h) Cold tolerance test in WT and KO mice post-HFD. N=5. For all data, mean+/- SEM (error bars). *$p<0.05$, (two-sided Student's t-test).
