# Supplementary material for: Telomerase Knockout in Myeloid Cells Predisposes Mice to Foam Cell Formation, Dyslipidemia, Lung Fibrosis, and Cardiac Dysfunction
Source: Aging Cell. 2026 Apr 16;25(4):e70490. doi: 10.1111/acel.70490 (PMC13086613; doi:10.1111/acel.70490)
Supplement: Supplementary file 3 — Figure S3: Metabolic dysfunction in LysM‐Tert KO mice fed an atherogenic diet. Mice were analyzed after atherogenic diet feeding for 6 weeks. (a) Body composition of WT and KO male and female mice. (b) Atherogenic diet consumption by WT and KO mice. (c) Glucose tolerance test in WT and KO mice. (d) Respiratory exchange ratio (RER) in WT and KO mice. (e) Plasma triglycerides in WT and KO mice. For (a–e), N = 5. (f) Representative cross sections of aortic arch from WT and KO 1‐year‐old males, stained with hematoxylin/eosin. Scale bar: 100 μm. Arrow: representative wall thickening in KO mice. Measurement of wall thickness at N = 25 random cross‐section points for N = 5 mice is quantified by ImageJ, on the right. Plotted are mean+/− SEM (error bars). *p < 0.05, (two‐sided Student's t‐test). [file ACEL-25-e70490-s003.pdf]

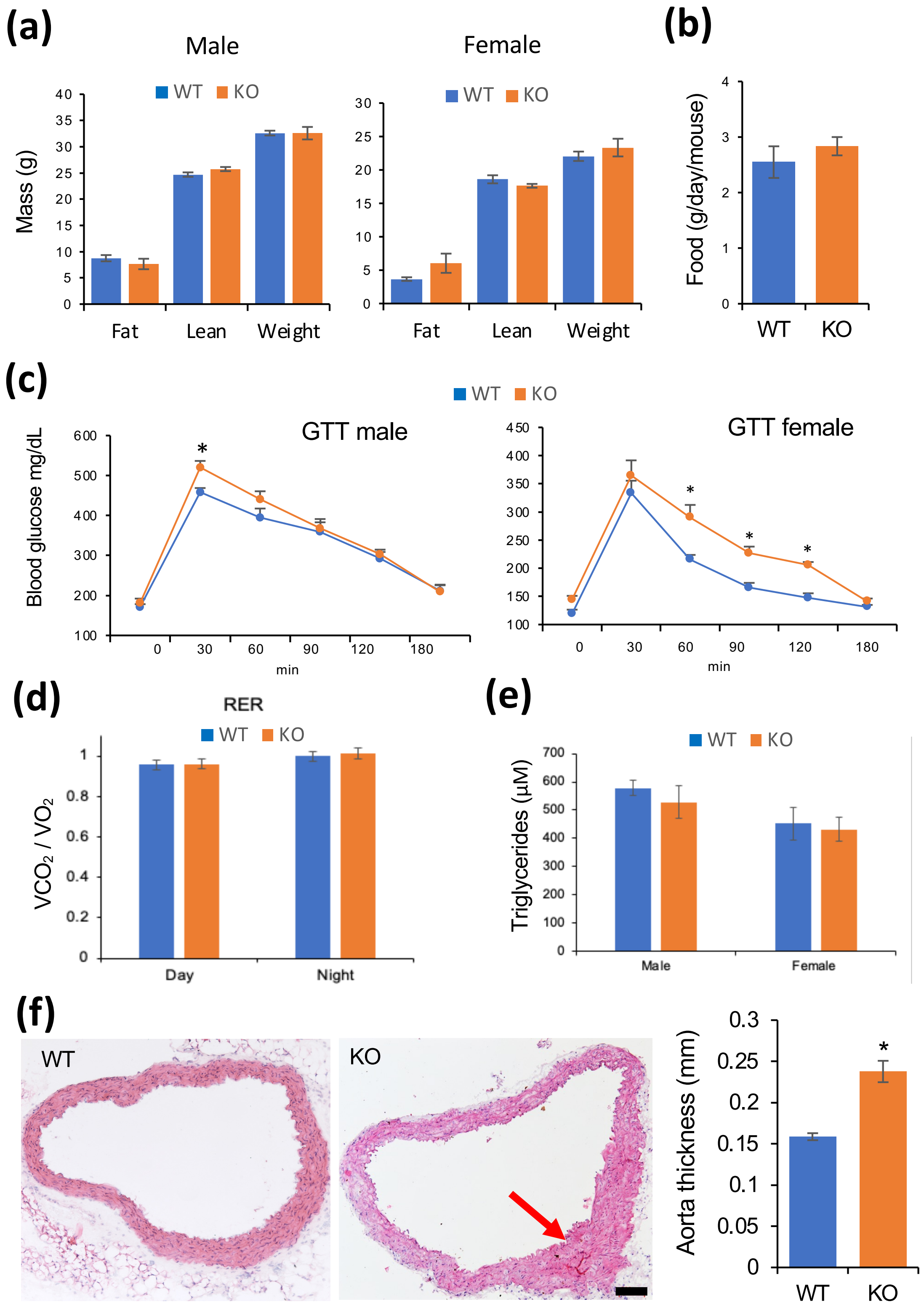

Figure S3 Metabolic dysfunction in LysM-*Tert* KO mice fed an atherogenic diet. Mice were analyzed after atherogenic diet feeding for 6 weeks. (a) Body composition of WT and KO male and female mice. (b) Atherogenic diet consumption by WT and KO mice. (c) Glucose tolerance test in WT and KO mice. (d) Respiratory exchange ratio (RER) in WT and KO mice. (e) Plasma triglycerides in WT and KO mice. For (a-e), N=5. (f) Representative cross-sections of aortic arch from WT and KO 1-year-old males, stained with hematoxylin/eosin. Scale bar: 100 µm. Arrow: representative wall thickening in KO mice. Measurement of wall thickness at N=25 random cross-section points for N=5 mice is quantified by ImageJ, on the right. Plotted are mean+/- SEM (error bars). *p<0.05, (two-sided Student's t-test).
