# Supplementary material for: Telomerase Knockout in Myeloid Cells Predisposes Mice to Foam Cell Formation, Dyslipidemia, Lung Fibrosis, and Cardiac Dysfunction
Source: Aging Cell. 2026 Apr 16;25(4):e70490. doi: 10.1111/acel.70490 (PMC13086613; doi:10.1111/acel.70490)
Supplement: Supplementary file 4 — Figure S4: AT abnormalities in LysM‐Tert KO mice. (a) IF with antibodies against GFP and RFP reveals a lower frequency of mG+ lineage cells (arrows) in VAT of KO male mice fed an atherogenic diet for 12 weeks. (b) IF with antibodies against CD68 and CD206 reveals a lower frequency of CD206+ macrophages (red arrows) in VAT of KO mice. IF with antibodies against perilipin‐1 and F4/80 reveals comparable adipocyte size in SAT of WT and KO mice. 4‐month‐old male mice fed an atherogenic diet were analyzed. Scale bar: 50 μm. (c) Flow cytometry analysis of VAT from MC‐Tert‐KO versus WT chow‐fed females (12 months old). Antibodies used: Cytek Aurora and FlowJo were used. [file ACEL-25-e70490-s002.pdf]

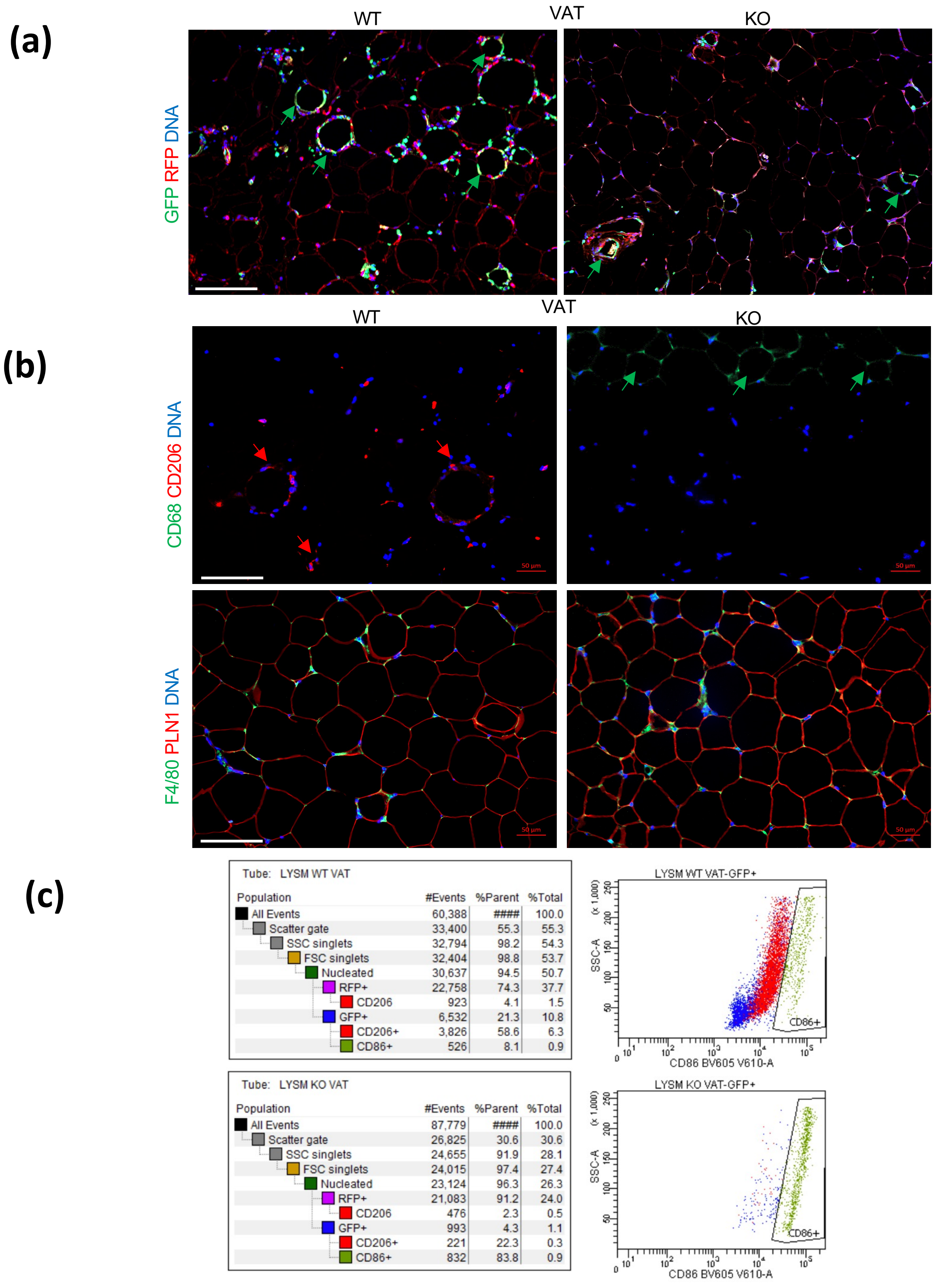

Tube: LYSM WT VAT

Population	#Events	%Parent	%Total
All Events	60,388	####	100.0
Scatter gate	33,400	55.3	55.3
SSC singlets	32,794	98.2	54.3
FSC singlets	32,404	98.8	53.7
Nucleated	30,637	94.5	50.7
RFP+	22,758	74.3	37.7
CD206	923	4.1	1.5
GFP+	6,532	21.3	10.8
CD206+	3,826	58.6	6.3
CD86+	526	8.1	0.9

Tube: LYSM KO VAT

Population	#Events	%Parent	%Total
All Events	87,779	####	100.0
Scatter gate	26,825	30.6	30.6
SSC singlets	24,655	91.9	28.1
FSC singlets	24,015	97.4	27.4
Nucleated	23,124	96.3	26.3
RFP+	21,083	91.2	24.0
CD206	476	2.3	0.5
GFP+	993	4.3	1.1
CD206+	221	22.3	0.3
CD86+	832	83.8	0.9

Figure S4 AT abnormalities in LysM-*Tert* KO mice**.** (a) IF with antibodies against GFP and RFP reveals a lower frequency of mG+ lineage cells (arrows) in VAT of KO male mice fed an atherogenic diet for 12 weeks. (b) IF with antibodies against CD68 and CD206 reveals a lower frequency of CD206+ macrophages (red arrows) in VAT of KO mice. IF with antibodies against perilipin-1 and F4/80 reveals comparable adipocyte size in SAT of WT and KO mice. 4-month-old male mice fed an atherogenic diet were analyzed. Scale bar: 50 µm. (c) Flow cytometry analysis of VAT from MC-*Tert*-KO vs WT chow-fed females (12 months old). Antibodies used:. Cytek Aurora and FlowJo were used.
