# Supplementary material for: Telomerase Knockout in Myeloid Cells Predisposes Mice to Foam Cell Formation, Dyslipidemia, Lung Fibrosis, and Cardiac Dysfunction
Source: Aging Cell. 2026 Apr 16;25(4):e70490. doi: 10.1111/acel.70490 (PMC13086613; doi:10.1111/acel.70490)
Supplement: Supplementary file 5 — Figure S5: Organ fibrosis in LysM‐Tert KO mice. Fixed sections from mice of the indicated ages fed chow were analyzed. (a) Trichrome staining reveals a lack of excessive fibrosis in the lungs of 6‐month‐old KO mice. (b) IF analysis reveals a higher frequency of mG+ macrophages (green arrows) in the liver of 12‐month‐old KO mice. (c) Trichrome staining reveals a lack of excessive fibrosis in the livers of 12‐month‐old KO mice. (d) Trichrome staining reveals a lack of excessive fibrosis in the hearts of 12 month‐old KO mice. Scale bar: 50 μm. Graphs: data quantification (mean +/− SEM) for a–d. N = 5. *p < 0.05 (two‐sided Student's t‐test). [file ACEL-25-e70490-s004.pdf]

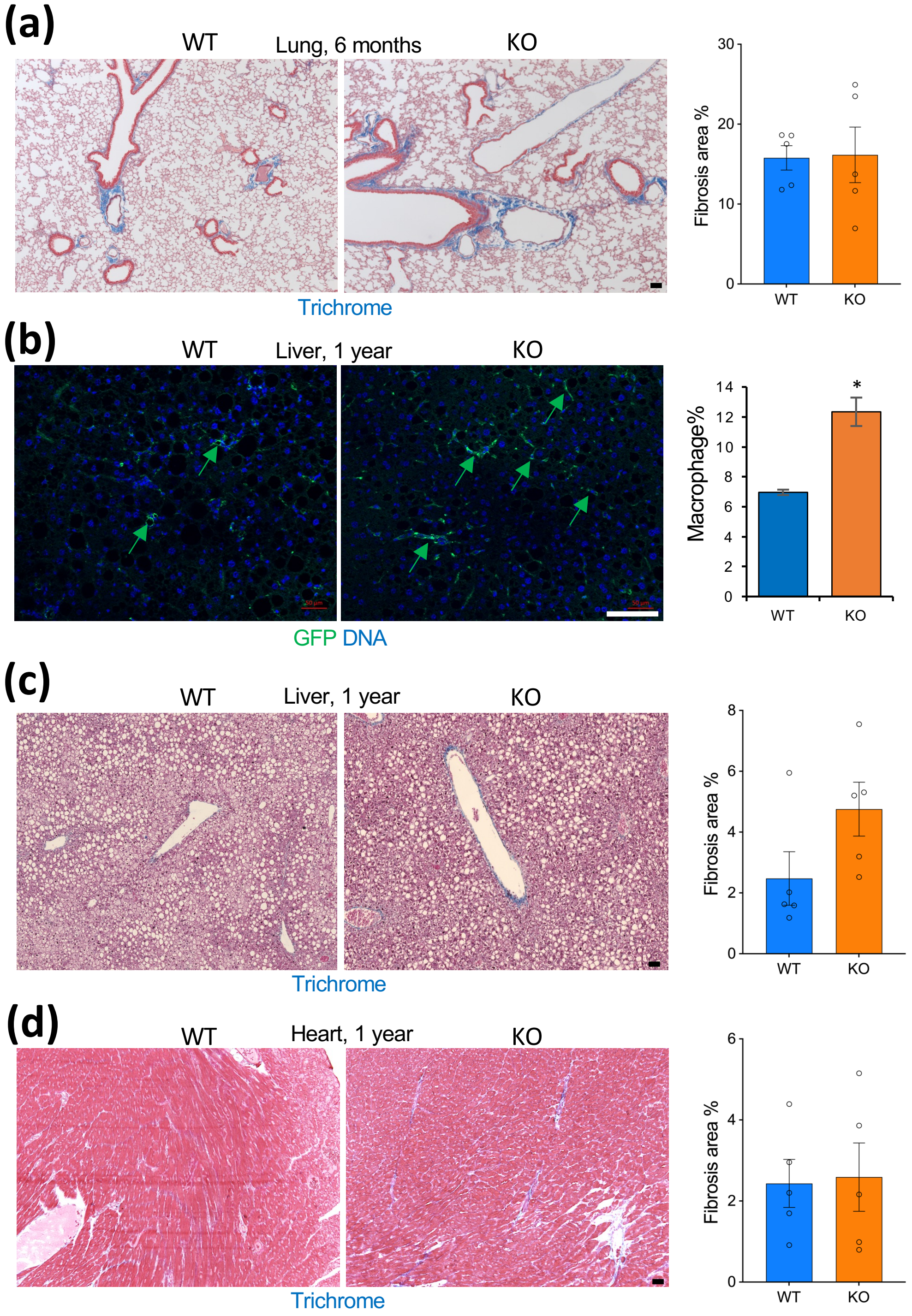

Figure S5 Organ fibrosis in LysM-*Tert* KO mice. Fixed sections from mice of the indicated ages fed chow were analyzed. (a) Trichrome staining reveals a lack of excessive fibrosis in the lungs of 6-month-old KO mice. b) IF analysis reveals a higher frequency of mG+ macrophages (green arrows) in the liver of 12-month-old KO mice. (c) Trichrome staining reveals a lack of excessive fibrosis in the livers of 12-month-old KO mice. (d) Trichrome staining reveals a lack of excessive fibrosis in the hearts of 12-month-old KO mice. Scale bar: 50 µm. Graphs: data quantification (mean+/- SEM) for a-d. N=5. *$p<0.05$ (two-sided Student's t-test).
