# Supplementary material for: Telomerase Knockout in Myeloid Cells Predisposes Mice to Foam Cell Formation, Dyslipidemia, Lung Fibrosis, and Cardiac Dysfunction
Source: Aging Cell. 2026 Apr 16;25(4):e70490. doi: 10.1111/acel.70490 (PMC13086613; doi:10.1111/acel.70490)
Supplement: Supplementary file 6 — Figure S6: Heart analysis. (a, b) 1‐year‐old WT and KO male mice fed atherogenic diet. (a) Hematoxylin/eosin of heart sections. (b) Heart sections subjected to IF with F4/80 antibodies. Arrows: increased frequency of macrophages in KO mice. IB4: isolectin B4 marking the endothelium. Scale bar: 50 μm. Graphs: data quantification (mean+/− SEM) for a‐d. N = 5. *p < 0.05 (two‐sided Student's t‐test). (c, d) Mice fed HFD for 1 year (also analyzed in Figure 6c,d). (c) Heart rate. (d) Ejection fraction (EF). (e) Cardiac output (CO) and stroke volume (SV) in 3 month‐old male mice fed chow. LV, left ventricle; RV, right ventricle. [file ACEL-25-e70490-s005.pdf]

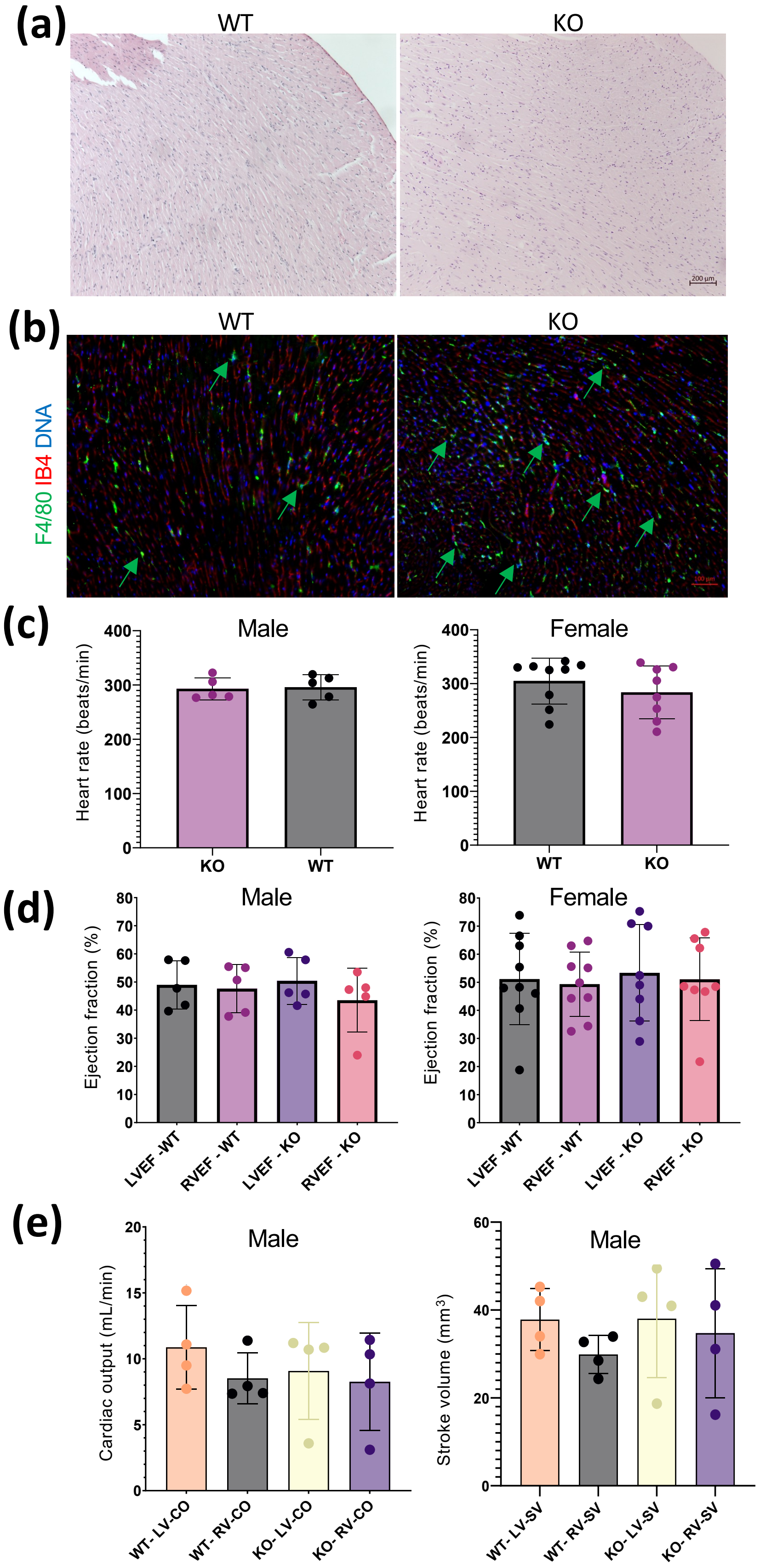

Figure S6 Heart analysis. (a-b): 1-year-old WT and KO male mice fed atherogenic diet. (a) Hematoxylin/eosin of heart sections. (b) Heart sections subjected to IF with F4/80 antibodies. Arrows: increased frequency of macrophages in KO mice. IB4: isolectin B4 marking the endothelium. Scale bar: 50µm. Graphs: data quantification (mean+/- SEM) for a-d. N=5. $*p<0.05$ (two-sided Student's t-test). (c-d): mice fed HFD for 1 year (also analyzed in Figure 6c-d). (c) Heart rate. (d) Ejection fraction (EF). (e) Cardiac output (CO) and stroke volume (SV) in 3-month-old male mice fed chow. LV: left ventricle; RV: right ventricle.
